# Capture Fluorocarbon and Chlorofluorocarbon from Air Using DUT‐67 for Safety and Semi‐Quantitative Analysis

**DOI:** 10.1002/advs.202308123

**Published:** 2024-01-19

**Authors:** Xiao‐Hong Xiong, Liang Song, Wei Wang, Hui‐Ting Zheng, Liang Zhang, Liu‐Li Meng, Cheng‐Xia Chen, Ji‐Jun Jiang, Zhang‐Wen Wei, Cheng‐Yong Su

**Affiliations:** ^1^ MOE Laboratory of Bioinorganic and Synthetic Chemistry GBRCE for Functional Molecular Engineering LIFM IGCME School of Chemistry Sun Yat‐Sen University Guangzhou 510006 China

**Keywords:** chlorofluorocarbon analysis, chlorofluorocarbon capture, gas adsorption and selectivity, gas separation, metal‐organic framework

## Abstract

Fluoro‐ and chlorofluorocabons (FC/CFCs) are important refrigerants, solvents, and fluoropolymers in industry while being toxic and carrying high global warming potential. Detection and reclamation of FC/CFCs based on adsorption technology with highly selective adsorbents is important to labor safety and environmental protection. Herein, the study reports an integrated method to combine capture, separation, enrichment, and analysis of representative FC/CFCs (chlorodifluoromethane(R22) and 1,1,1,2‐tetrafluoroethane (R134a)) by using the highly stable and porous Zr‐MOF, DUT‐67. Gas adsorption and breakthrough experiments demonstrate that DUT‐67 has high R22/R134a uptake (124/116 cm^3^ g^−1^) and excellent R22/R134a/CO_2_ separation performance (IAST selectivities of R22/CO_2_ and R134a/CO_2_ ranging from 51.4 to 33.3, and 31.1 to 25.8), even in rather low concentration and humid conditions. A semi‐quantitative analysis protocol is set up to analyze the low concentrations of R22/R134a based on the high selective R22/R134a adsorption ability, fast adsorption kinetics, water‐resistant utility, facile regeneration, and excellent recyclability of DUT‐67. In situ single‐crystal X‐ray diffraction, theoretical calculations, and in situ diffuse reflectance infrared Fourier transform spectra have been employed to understand the adsorption mechanism. This work may provide a potential adsorbent for purge and trap technique under room temperature, thus promoting the application of MOFs for VOCs sampling and quantitative analysis.

## Graphical abstract

A semi‐quantitative analysis method, integrating capture, separation, enrichment, and analysis into one, is developed for highly efficient detection and analysis of low concentrations of chlorodifluoromethane(R22) and 1,1,1,2‐tetrafluoroethane (R134a)) under room temperature by using the highly stable and porous Zr‐MOF, DUT‐67, bearing plenty of coordinated H_2_O/OH^−^, as a solid adsorbent.

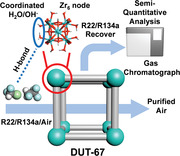

## 1 Introduction

The Fluoro‐ and chlorofluorocabons (FC/CFCs) are widely used in industry,^[^ ^1^ ^]^ resulting in thousand times greenhouse effect of carbon dioxide due to their high global warming potential (GWP). Moreover, they are toxic^[^ ^2^ ^]^ as volatile organic compounds (VOCs), thereof must be carefully handled to prevent leakage during production and usage.^[^ ^3^ ^]^ However, it is unavoidable to release FC/CFCs into the atmosphere due to human activities, and trace FC/CFCs can cross the air‐sea interface to dissolve in the surface of seawater.^[^ ^4^ ^]^ As a result, the oceanic FC/CFCs can be used as the transient tracers to trace water‐mass motion and mixing, quantify the ocean uptake of anthropogenic CO_2_, assess ocean circulation and climate models, and estimate melting rate of polar ice shelves and the water production rate from ice shelve, etc.^[^ ^5^ ^]^ Thus, the recovery and quantitative analysis of low concentrations of FC/CFCs in air and seawater are very important for human health, environment protection, as well as marine science research. However, since the concentration of FC/CFCs is extremely low in atmosphere and seawater, usually at a ng L^−1^ level,^[^ ^4^, ^5^, ^6^ ^]^ it is difficult to directly and accurately quantify FC/CFCs in air and seawater, requiring instruments with high sensitivity and low detection limit.

To quantitatively detect and analysis FC/CFCs in the atmosphere and seawater, the samples containing trace FC/CFCs always need to be preconcentrated. A purge and trap gas‐chromatographic system is widely used, but its trap‐processes are complicated.^[^ ^4^ ^]^ Typically, the highly pure N_2_ is first injected into the seawater sample to drive out the dissolved FC/CFCs, and the gas mixture flows through a packing column filled with solid adsorbents to capture and enrich FC/CFCs at low temperature (<−30 °C). Next, the packing column is heated to desorb the accumulated FC/CFCs, and finally reaching to a concentration that can be measured. For this detection method, the performance of solid adsorbents is crucial, deciding the accuracy of analysis results. So far, Porapak Q, Porasil C, Unibeads 2S, Porapak T, mesh 13X, 5A, and 4A have been reported as the solid adsorbents used in the purge and trap technique to detect HC/CFCs in seawater and the atmosphere.^[^ ^4^, ^7^ ^]^ Among these, owing to their high stability, high adsorption affinity, and easy availability, molecular sieves, like mesh 13X, 5A, and 4A, are commonly used as solid adsorbents to trap or adsorb HC/CHCs,^[^ ^8^ ^]^ but they lack sufficient trap capacity and selectivity.^[^ ^8^, ^9^ ^]^ On the other hand, large‐scale FC/CFCs recycling and separation in industrial resort to cryogenic separation, which suffers from high energy penalties, expenses, and safety risks.^[^ ^10^ ^]^ Therefore, developing new types of porous materials with high adsorption selectivity and capacity of FC/CFCs is demanded for both FC/CFCs decontamination and enrichment at a low concentration, and larger scale FC/CFCs separation and reclamation.^[^ ^11^ ^]^

Metal‐organic frameworks (MOFs) have been developed as a type of excellent crystalline porous materials constructed from inorganic nodes and organic linkers, showing promising potentials in gas storage/separation, heterogeneous catalysis, chemical sensing, drug delivery, and other areas.^[^ ^12^ ^]^ Compared to other FC/CFC sorbents like activated carbons and zeolites, the large surface areas and high pore volumes of MOFs can significantly increase the total uptake of FC/CFCs.^[^ ^13^ ^]^ Their tunable pore sizes and functioning frameworks benefit the gas adsorption selectivity owing to a sieving effect and abundant host–guest interactions. Additionally, their physisorption nature also ensures fast and energy‐efficient adsorption and regeneration.^[^ ^1^, ^13^, ^14^ ^]^ Especially for Zr‐MOFs, they possess extraordinary thermal and chemical stability for feasible FC/CFCs sorption applications. For example, Motkuri and Thallaplly studied the adsorption behaviors and their breakthrough separation simulations of a series of fluorocarbons using microporous and hierarchically mesoporous MOFs.^[^ ^15^ ^]^ Liu group reported UiO‐66 for separation of the binary (R22/R32 and R32/R125) and ternary (R32/R125/R134a) FC/CFC mixtures with high selectivities and capacities.^[^ ^11a^ ^]^ Our group has been interested in studying FC/CFCs adsorption and separation by using stable MOFs.^[^ ^16^ ^]^ DUT‐67 is constructed with 8‐connnected Zr_6_‐nodes and *2,5*‐thiophenedicarboxylate linkers (TDC), possessing large porosity and high stability.^[^ ^17^ ^]^ Its original and functionalized versions have been studied for photocatalysis,^[^ ^18^ ^]^ water adsorption,^[^ ^19^ ^]^ chemical sensing,^[^ ^20^ ^]^ and other applications.^[^ ^21^ ^]^ We propose that DUT‐67 may be a good FC/CFCs adsorbent because of its large pores and Zr_6_‐nodes featuring an abundance of OH^−^/H_2_O groups, which can interact with F and Cl atoms through O―H…F or O―H…Cl forces to selectively grab FC/CFCs, thus increasing the adsorption affinity and selectivity toward FC/CFCs.

Herein, we utilize a highly stable and porous Zr‐MOF, DUT‐67, for selective capture and quantitatively determination of the representative R22 and R134a (CHClF_2_ and CH_2_FCF_3_, representing FC/CFCs with/without Cl) from air. We performed a series of adsorption experiments to obtain both the heat of adsorption (Q_st_) and the idea adsorption solution theory (IAST) selectivity, demonstrating that DUT‐67 has high selectivity for R22/R134a over CO_2_. The breakthrough experiments further confirm that DUT‐67 can effectively capture low concentrations of R22 and R134a in both dry and humid air with good recyclability. Through performing trap and purge experiments under room temperature, we have successfully realized direct capture and semi‐quantitative determination of low concentrations of FC/CFCs. The R22 and R134a binding sites in DUT‐67 have been determined by virtue of the in situ single crystal X‐ray diffraction (SCXRD) and in situ diffuse reflectance infrared Fourier transform (DRIFT‐IR) spectra, further corroborated by the theoretical calculation results, in which the OH^−^/H_2_O groups on the Zr_6_ nodes play a critical role in selectively capturing R22 and R134a by forming multiple Cl/F··· H−O and O···H−C hydrogen bonds. This work provides a potential porous solid adsorbent candidate for the purge and trap technique to quantitative analyze FC/CFCs in seawater or air, and may promote the application of MOF materials in the field of VOCs sampling and quantitative analysis.

## 2 Results and Discussion

### 2.1 Synthesis and Characterization

**Figure 1:**
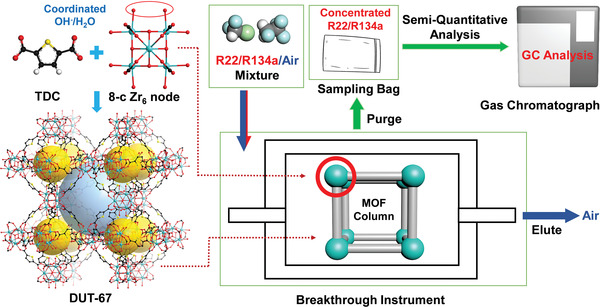
Diagram of a system integrating adsorption, separation, enrichment, and GC analysis for low concentrations of FC/CFCs by using DUT‐67 as a column adsorbent. The gold/pale blue spheres stand for pores with the diameters of 7/11 Å, respectively. Color scheme: black, C; red, O; yellow, S; aqua, Zr. Hydrogen atoms are omitted for clarity.

The pure DUT‐67 was synthesized through an optimized procedure from our previous work,^[^ ^22^ ^]^ except replacing the modulator acetic acid with formic acid and the solvent *N,N’*‐dimethylformamide (DMF) with *N,N’*‐dimethylacetamide (DMAC) reported by Senkovska group.^[^ ^17^ ^]^ As certified by the neat powder X‐ray diffraction (PXRD) patterns (Figure S1, Supporting Information), this modified synthetic route provided highly crystalline product for easy scale‐up preparation.^[^ ^23^ ^]^ The thermogravimetric analyses (TGAs) showed that DUT‐67 is stable up to 220 °C (Figure S2, Supporting Information), further confirmed by the variable‐temperature PXRD measurements (Figure S3, Supporting Information). The water adsorption isotherms for three rounds on DUT‐67 are comparable, confirming that its internal surface remained intact toward repeating water molecule attack (Figure S4, Supporting Information). The integrity of the framework was further confirmed by PXRD after the third water adsorption (Figure S5, Supporting Information). The samples were also soaked in 1 m NaOH, 6 m HCl, 12 m HCl, and boiling water for 24 h, and seawater for one month, to assess the chemical stability of DUT‐67. The PXRD patterns measurements demonstrate that DUT‐67 possesses a good tolerance against harsh conditions (Figure S6, Supporting Information), affirming its excellent chemical stability.^[^ ^17^ ^]^ The porosity of the activated DUT‐67 was evaluated by nitrogen adsorption measurement at 77 K (Figure S7, Supporting Information). The Brunauer−Emmett−Teller (BET) surface area and total pore volume are calculated to be 1300 m^2^ g^−1^ and 0.48 cm^3^ g^−1^ (Figures S8 and S9, Supporting Information), respectively. The pore size distribution analysis revealed that the framework contains two types of pores with diameters of 0.58 and 1.12 nm, respectively, which is consistent with its single‐crystal structure analysis results (**Figure** **1**; Figure S9, Supporting Information). The water adsorption isotherms exhibited two steps, indicating the successive filling of the pores with different sizes (Figure S4, Supporting Information).

### 2.2 Gas Adsorption, Separation, and Recovery

**Figure 2:**
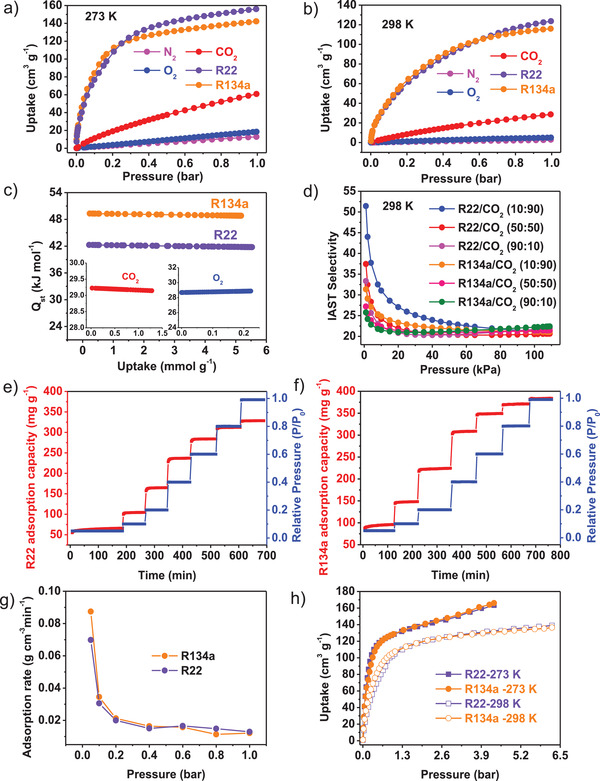
R22, R134a, CO_2_, O_2_, and N_2_ adsorption isotherms for DUT‐67 at a) 273 K and b) 298 K. c) Q_st_ of the adsorbed gases as a function of the surface coverage. d) IAST selectivity for different gas mixtures. Adsorption kinetic curves of DUT‐67 for e) R22 and f) R134a. g) The adsorption rates of R22 and R134a versus pressure for DUT‐67. h) High‐pressure R22 and R134a adsorption isotherms of DUT‐67.

The single component gas adsorptions of R22, R134a, CO_2_, O_2_, and N_2_ were measured at 273 and 298 K, respectively (**Figure** **2**a,b). Under 298 K and 1 bar, the uptakes of R22 (124 cm^3^ g^−1^) and R134a (116 cm^3^ g^−1^) are much higher than those of CO_2_ (39 cm^3^ g^−1^), O_2_ (5 cm^3^ g^−1^), and N_2_ (3 cm^3^ g^−1^). Notably, even at very low pressure (0.1 bar), DUT‐67 still has excellent R22 and R134a uptake capacities. The R22 and R134a uptake capacities at 298 K and 1 bar are lower than those of the record holder, LIFM‐67 (249.8 and 249.5 cm^3^ g^−1^),^[^ ^9a^ ^]^ but outperforming those of most reported MOFs and molecular sieves (Table S1, Supporting Information).^[^ ^8^, ^11^, ^14^, ^16^ ^]^ The consecutive (ten cycles) R22 and R134a adsorption‐desorption measurements of DUT‐67 at 298 K were executed to demonstrate its excellent durability and recyclability (Figure S10, Supporting Information).

The adsorption kinetic results indicate that the adsorption rates of R22 and R134a decrease along adsorption pressure following a pseudo‐second‐order process (Figure 2e–g; Table S2, Supporting Information), and the highest rate is reached at the lowest adsorption partial pressure, suggesting that DUT‐67 has high potential to rapidly adsorb R22 and R134a at low concentration. The heats of adsorption (Q_st_) for these gases have been calculated based on adsorption data under 273 and 298 K, confirming that the Q_st_ values of R22 (42.3 kJ mol^−1^) and R134a (49.3 kJ mol^−1^) at zero coverage are much higher than those of CO_2_ (29.2 kJ mol^−1^) and O_2_ (28.7 kJ mol^−1^) (Figure 2c; Figures S11–S14, Supporting Information). Because of the large uptake difference between R22/R134a and O_2_/N_2_, it is no doubt that the selectivities of R22/R134a versus O_2_/N_2_ will be reasonably high. The R22/CO_2_ and R134a/CO_2_ selectivities have been calculated according to the ideal adsorption solution theory (IAST), which clearly shows abrupt increase at low pressure (Figure 2d). It is well known that the FC/CFCs leakage may cause relatively high concentration in isolated local environments under industrial conditions, and CO_2_ will be compete with FC/CFCs for adsorption, so we compare the selectivity of R22/CO_2_ and R134a/CO_2_ at 298 K and 1 kPa, which gives the R22/CO_2_ and R134a/CO_2_ selectivities ranging from 51.4 to 33.3, and 31.1 to 25.8, respectively, with varied volumetric ratios (10:90; 50:50; 90:10). These results imply that DUT‐67 has a potential for selectively capturing R22 and R134a from air. To determine the saturated adsorption capacity of DUT‐67, the high‐pressure R22 and R134a adsorption isotherms at 273 and 298 K have been measured, indicating that DUT‐67 show comparable R22 and R134a uptake capacities at 273 (163 and 166 cm^3^ g^−1^ at 4.3 bar) and 298 K (139 and 137 cm^3^ g^−1^ at 6.23 bar), respectively (Figure 2h).

**Figure 3:**
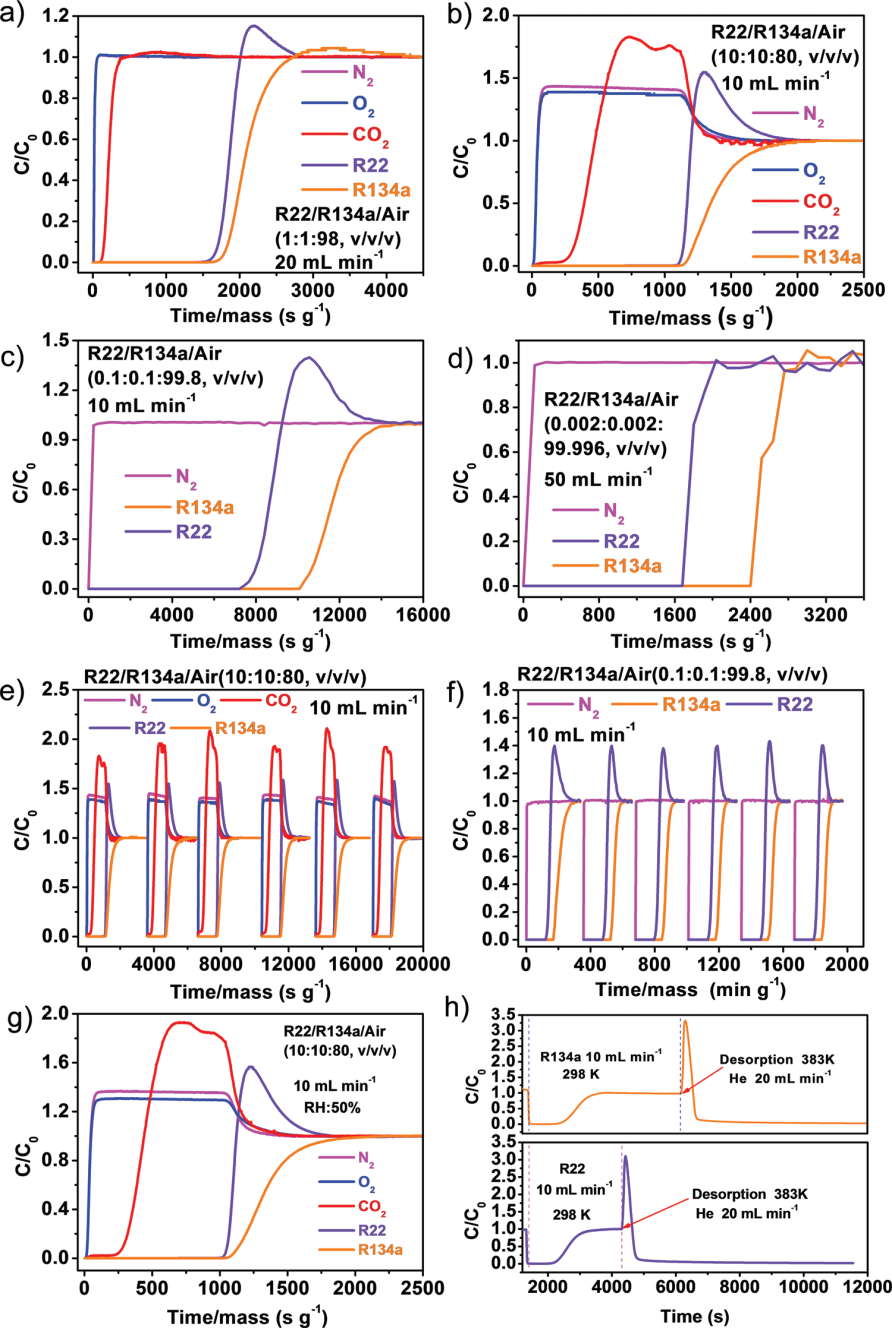
Breakthrough tests of R22/R134a/air mixtures with various volumetric ratios by DUT‐67. a) 1:1:98; b) 10:10:80; c) 0.1:0.1:99.8; d) 0.002:0.002:99.996. e,f) Cycling breakthrough tests for DUT‐67. g) Comparison of the dynamic breakthrough separation performance of DUT‐67 at 298 K under dry and 50% humid conditions. h) The capture‐desorption curves of pure R22 and R134a.

To evaluate the practically selective adsorption performance, dynamic breakthrough experiments have been performed under ambient conditions, where the R22/R134a/air mixtures with various volumetric ratios flowed through the packing column at a fixed total flow rates of 10, 20, or 50 mL min^−1^ (**Figure** **3**). First, a R22/R134a/air (1:1:98, v/v/v) mixture with a total flow rate of 20 mL min^−1^ was tested (Figure 3a). As expected, O_2_ and N_2_ eluted almost immediately without little adsorption, then CO_2_ eluted at ≈80 s g^−1^. R22 and R134a were effectively captured in the column before their saturated sorption and breakthrough (1465/1600 s g^−1^ for R22/R134a). Second, a R22/R134a/air (10:10:80, v/v/v) mixture with a total flow rate of 10 mL min^−1^ was tested (Figure 3b). Similarly, O_2_, N_2_ and CO_2_ gases all eluted at the beginning, and DUT‐67 could selectively capture R22 and R134a in such high concentration, which broke through at 842 and 1180 s g^−1^, respectively. The capture amounts of R22 and R134a by DUT‐67 were calculated to be 0.2651 and 0.3072 mmol g^−1^ based on a single breakthrough curve with the R22/R134a/air (1:1:98, v/v/v) mixture at a flow rate of 20 mL min^−1^, and 0.7478 and 1.0341 mmol g^−1^ based on a single breakthrough curve with the R22/R134a/air (10:10:80, v/v/v) mixture at a flow rate of 10 mL min^−1^, respectively. It should be mentioned that these two breakthrough curves were collected using mass spectrometry as the detector; unfortunately, the MS detector is not suitable to analyze R22/R134a with even lower concentrations, due to complex molecule fractions in the mass spectrometry background produced during ionization.

To measure the breakthrough curves for the mixed gases containing rather low R22/R134a concentrations, a system using gas chromatograph (GC) as the monitor was applied instead. Due to the limit of detection (LOD) of GC and a lack of gas chromatographic column that can simultaneously separate and record the signals of R22/R134a/N_2_/CO_2_/O_2_ in a short retention time, we were not able to monitor the signals of O_2_ and CO_2_ in real‐time. Therefore, a R22/R134a/air (0.1:0.1:99.8, v/v/v) mixture with a total flow rate of 10 mL min^−1^ was tested. As shown in Figure 3c, N_2_ was still the first gas to elute. R22 and R134a broke through at 7440 and 10 320 s g^−1^, respectively. The capture amounts are 0.05708 and 0.08711 mmol g^−1^ for R22 and R134a, respectively. Finally, considering the fact that the concentration of FC/CFCs in seawater and atmosphere is at ng L^−1^ level, a gases mixture of R22/R134a/air (0.002:0.002:99.996, v/v/v) with extremely low FC/CFC concentration has been tested with a total flow rate of 50 mL min^−1^ (Figure 3d). The curves of R22 and R134a become unsmooth because the integrated peak areas of R22 and R134a at 0.002% are close to the LOD of GC; nevertheless, the convincing breakthrough results were obtained. The breakthrough time and capture amounts of R22/R134a are 1800 and 2520 s g^−1^ (30 and 42 min g^−1^), and 0.001428 and 0.01937 mmol g^−1^, respectively. These results coincide well with the Q_st_ values and the IAST selectivity calculations, confirming that DUT‐67 has stronger affinity toward R22 and R134a than other air components to selectively adsorb them. The recyclability of DUT‐67 for gas separation has been evaluated by cycling breakthrough experiments for two gas mixtures of R22/R134a/air (10:10:80, v/v/v) and R22/R134a/air (0.1:0.1:99.8, v/v/v) (Figure 3e,f), which showcase quite similar breakthrough curves during six cycles of experiments, demonstrating its excellent durability for R22 and R134a capture.

Since the water vapor is ubiquitous and often severely influences the gas adsorption performance of MOFs, we also tested the FC/CFCs capture by DUT‐67 under humid air, using the gas mixtures of R22/R134a/air (10:10:80, v/v/v) under 50 and 80% relative humidity (RH) with the total flow rate of 10 mL min^−1^ (Figure 3g; Figure S15, Supporting Information). The breakthrough orders of the gases have not been changed, namely, N_2_/O_2_ > CO_2_ > R22 > R134a. The breakthrough times for CO_2_, R22, and R134a are still comparable with those under dry air mixture, and the capture amounts for R22 and R134 are just slightly lower than those in dry air, manifesting good adsorption performance of DUT‐67 to resist water deterioration for a real application. Moreover, rapid desorption of R22 and R134 from the packing column has been testified. A complete adsorption‐desorption curves of pure R22 and R134a were collected with breakthrough instrument (Figure 3h; Figure S16, Supporting Information), illustrating that the R22 and R134a gases captured in the packing column can be quickly desorbed by helium purge at 110 °C.

### 2.3 Semi‐Quantitative Analysis

Prompted by the outstanding ability of DUT‐67 involving the capture of extremely low concentrations of FC/CFCs, together with the high adsorption rate at low partial pressure, rapid desorption kinetics, resistance to water vapor interference, easy regeneration and good recyclability, we tried to establish a system to integrate adsorption, enrichment, separation, and analysis processes for FC/CFCs (Figure 1). The quantitative analysis based on the desorbed analyte on the DUT‐67 packed column includes the following steps: 1) Working curves preparation. The job plots correlating the GC peak areas and R22/R134a amounts were determined by using a commercially standard R22/R134a/air mixture (Figures S17 and S18, Supporting Information). 2) Desorption process check. We selected the low concentrations of gas mixture, R22/R134a/air (0.002:0.002:99.996, v/v/v), to flow through the DUT‐67 packed column with a total flow rate of 50 mL min^−1^ for 30 min. Then the column is heated to 100 °C, followed by He purge with a flow rate of 5 mL min^−1^ until the target gas signals disappeared completely (Figure S19, Supporting Information). This step provides the information on how much time is needed to completely desorb the target gases on the column. 3) Blank breakthrough calibration. The calibration factors for R22/R134a content calculations were obtained from 48 times repeating measurements of R22/R134a concentrations directly by GC analysis of 1 mL vent gas after breakthrough process without DUT‐67 adsorption (Table S3, Supporting Information). 4) FC/CFC enrichment. A same gas mixture was flowed through the DUT‐67 packed column with a total flow rate of 50 mL min^−1^ for 30 min. After stopping the gas flow, one gas sampling bag was connected to the column outlet. The same desorption procedure was performed, i.e., heating the column to 100 °C and then purging it with He. After 60 min, 300 mL gas was collected. 5) GC analysis. A micro‐syringe was used to gather 250 µL sample from the bag and inject it into the GC. The obtained peak area was used to calculate the concentrated R22/R134a contents in the gas sampling bag. Then the total capture amount and gas concentrations in the original gas mixture can be calculated from the equations described in the Experimental Section.

We have repeated this adsorption‐desorption‐analysis process for three times to check the quantitative analytical results of R22/R134a (Tables S4 and S5, Supporting Information). The average concentrations of R22 and R134a are calculated to be 20.87 and 21.93 ppm, respectively, close to the theoretical value (20 ppm) with relative errors of 4.17 and 8.80% (Table S6, Supporting Information). The standard deviations (SDs) and relative standard deviations (RSDs) are 0.17/0.87 and 0.83/3.98% for R22 and R134a analyses, respectively. These results suggest that this analytical method can provide a semi‐quantitative determination of the low concentrations of FC/CFCs, although the combined processes of the gas breakthrough, desorption, and collection may bring in unavoidable systematic errors. Considering the practical difficulty in accurate quantification of FC/CFC concentrations in real atmosphere and seawater, which inevitably needs selective capture and enrichment, this semi‐quantitative method may be useful owing to its features of easy combination of FC/CFC capture, separation, enrichment, and analysis, offering a convenient way to monitor the content of FC/CFCs at low concentration quickly.

### 2.4 Gas Binding Mechanism

**Figure 4:**
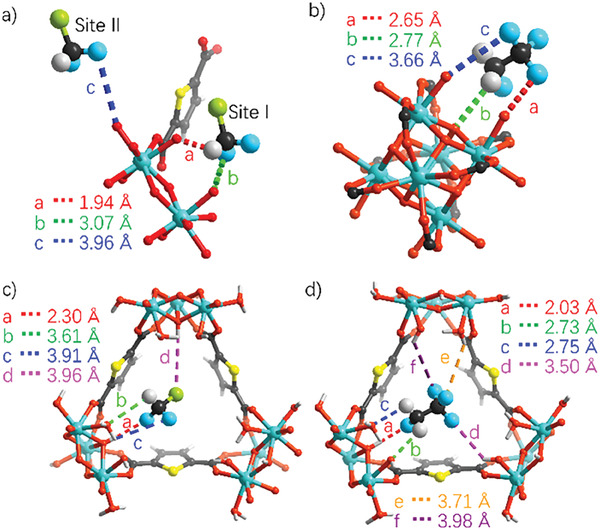
The preferential binding sites of a) R22 and b) R134a in DUT‐67 pores determined by in situ single‐crystal X‐ray diffraction. DFT calculated preferential adsorption sites of c) R22 and d) R134a in optimized DUT‐67. The HBs are labeled as dotted lines, of which a/b refer to X…Y distances and c/d refer to H…Y distances.

To reveal the mechanism of R22/R134a adsorption, the single‐crystals of DUT‐67 capturing R22/R134a molecules were prepared, and their structures were determined by in situ single‐crystal X‐ray diffraction analysis (in situ SCXRD) (Table S7, Supporting Information). For R22, two preferential adsorption sites I and II are determined to locate near the triangle apertures of the small and large pores, respectively (**Figure** **4**a,b). Although the H atoms of OH^−^/H_2_O groups on the Zr_6_‐node cannot be defined by X‐ray diffraction, and the R22/R134a molecules are disordered over fractional positions, the hydrogen bonds (HBs) still can be discerned. R22 at site I is hold by two O‐H···F and C‐H···O HBs to bind with OH^−^/H_2_O groups on one Zr_6_‐node; while R22 at site II is hold by one O‐H···F HB to connect to the OH^−^/H_2_O group on the same Zr_6_‐node. For R134a, only one preferential adsorption site is found in the small pore of DUT‐67. The R134a is hold by three O‐H···F and C‐H···O HBs to interact with the OH^−^/H_2_O groups on the Zr_6_‐node. These gas‐framework interactions account for the high Q_st_ of R22 and R134a, underpinning preferential selectivity for FC/CFCs adsorption, similar to the H_2_S adsorption.^[^ ^24^ ^]^

To further elucidate the binding types of R22/R134a with the framework, the grand canonical Monte Carlo (GCMC) simulations and first‐principle density functional theory (DFT) calculations were performed. The density distribution mappings show that the favorable adsorption sites are located at the cavity window positions near Zr_6_‐nodes (Figure S20, Supporting Information). The DFT calculation results provide information for the primary adsorption site. For R22‐loaded DUT‐67, the gas molecule locates at the triangle window and hold to two Zr_6_‐node by four HBs ranging from 2.30 to 3.96 Å (Figure 4c,d). Similarly, R134a molecules are also at the triangle window and bind to three Zr_6_‐nodes via four HBs ranging from 2.03 to 3.98 Å. The adsorption locations of R22 and R134a obtained by DFT calculation are basically consistent with those determined by the in situ SCXRD (Figure S21, Supporting Information), giving the averaged location centered in the distributed positions in the single‐crystal structures, and falling in the potential binding regions revealed by the GCMC calculated adsorption density distribution mapping (Figure S20, Supporting Information). In addition, DFT calculations give the adsorption energies (ΔE) following the same trend as Q_st_, i.e., Δ*E*_R134a_ (34.31 kJ mol^−1^) > Δ*E*_R22_ (26.31 kJ mol^−1^, Table S8, Supporting Information).

**Figure 5:**
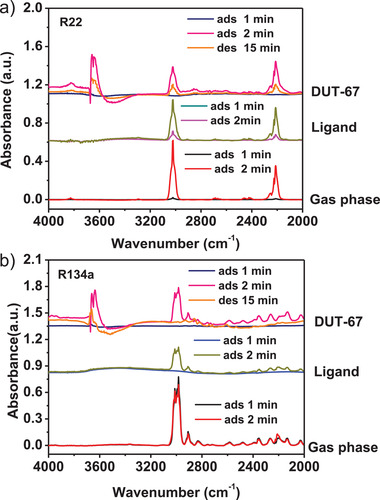
In situ DRIFT spectra of DUT‐67 at various loading of a) R22 and b) R134a.

More R22/R134a binding information has been obtained from the in situ diffuse reflectance infrared Fourier transform (DRIFT) spectroscopy. The vibration of −OH group in the activated DUT‐67 was observed at 3639 cm^−1^ (Figure S22, Supporting Information), which was compared with the spectra recorded in R22 and R134a atmosphere under transmission mode. As shown in **Figure** **5**, new adsorption bands in 3500–3671 cm^−1^ region were observed after subtraction of the original sample spectra and the background when R22 or R134a were injected. The adsorption peak intensities increased along the gas injection time while decreased when the samples were desorbed, reflecting the influence on the −OH vibrations by the −OH···F or −OH···Cl hydrogen binding interactions.

## 3 Conclusion

In conclusion, we have demonstrated that the DUT‐67, featuring excellent physicochemical stability, presents high R22 and R134a selective adsorption performance under both low and high pressures, and both dry and humid conditions. The dynamic breakthrough experiments conducted for R22/R134a/air mixtures with various volumetric ratios, especially with very low FC/CFCs concentration, reveal excellent performance of DUT‐67 to separate and reclaim R22/R134a from air with good recyclability and durability. A semi‐quantitative analysis protocol has been proposed to determine the concentration of R22/R134a based on the outstanding adsorption‐desorption kinetics for the low concentrations of FC/CFC enrichment. The preferential R22/R134a adsorption mechanism has been elucidated by in situ SCXRD, theoretical calculations, and in situ DRIFT, revealing the key importance of the coordinated OH^−^/H_2_O groups on the Zr_6_‐node for R22/R134a capture. Noteworthily, in comparison with the traditional yet widely applied purge and trap gas‐chromatographic system using a cold trap and 13X zeolite as the solid adsorbent, our proposed semi‐quantitative analysis method using DUT‐67 as an R22 and R134a reservoir under room temperature presents some advantages, involving high R22 and R134a enrichment capacity (124 and 116 cm^3^ g^−1^ at 298 K and 1 bar), low CO_2_ capture capacity (39 cm^3^ g^−1^ at 298 K and 1 bar), outstanding R22/R134a/air separation, good hydrophobicity in the low‐pressure region, fast adsorption–desorption kinetics, simplified equipment system, as well as energy and cost efficiency, thereby resulting in the rapid and highly efficient detection of low concentrations of FC/CFCs in air or oceans.^[^ ^9b^ ^]^ This work provides a paradigm for how to design and construct suitable MOF candidates to deal with challenging gas separation and detection in an efficient and easy‐handling way.

## 4 Experimental Section

### DUT‐67 Synthesis

DUT‐67 was prepared following the previously modified procedure.^[22]^See characterization details in Supporting Information.

### In Situ Single‐Crystal X‐Ray Diffraction

Two 10 mL plastic centrifuge tubes were immersed into the liquid nitrogen for several minutes. Then R22 and R134a were injected into the plastic centrifuge tubes until the gases liquefied, respectively. The gas injections were stopped and the plastic tubes were kept immersed in liquid nitrogen. Then the activated DUT‐67 was added into the two plastic centrifuge tubes and sealed, immersing the crystals in R22/R134a liquid. After 10 min, the tubes were opened to let the liquid vaporize completely. Finally, the crystals were taken out for data collection using single crystal diffractometer at 240 K.

### Semi‐Quantitative Analysis of R22/R134a

*Working Curves*: A series of gas samples (25, 50, 100, 150, 200, and 250 µL) from a mixture of R22/R134a/air (0.1:0.1:99.8, v/v/v) were injected into GC, and the peak areas of R22 and R134a were recorded. For every sample, the peak areas were measured for four times to obtain the average value. The R22/R134a contents versus peak areas were plotted to give the working curves (Figures S17 and 18, Supporting Information).

*Desorption Time*: A gas mixture of R22/R134a/air (0.002:0.002:99.996, v/v/v) was flowed through the DUT‐67 column in a total flow rate 50 mL min^−1^ for 30 min. Afterward, the column was heated to 100 °C and purged by He with a 5 mL min^−1^ flow rate until no R22/R134a were detectable. The desorption process was determined to complete in 60 min (Figure S19, Supporting Information).

*R22/R134a Content Calibration*: The same gas mixture was flowed through a blank packing column without DUT‐67 to directly reach to the sampling port of the GC. 1 mL of the vent gas was introduced into GC, and the relative peak areas of R22/R134a were recorded. The GC sampling and detecting process was repeated for 48 times in every 2 min, giving the calibration factor *R* for R22/R134a content calculations (Table S3, Supporting Information).

*Gas Enrichment and Collection*: An analytic test was performed with the low FC/CFC concentration mixture of R22/R134a/air (0.002:0.002:99.996, v/v/v), which was flowed through the DUT‐67 packed column with a total flow rate (*v*_gas_) of 50 mL min^−1^ for 30 min (*t*_gas_). Instead of connecting to GC, a gas sampling bag was installed at the outlet, and the column was heated to 100 °C and purged by He with a flow rate (*v*_He_) of 5 mL min^−1^ for 60 min (*t*_He_). This adsorption–desorption process was repeated for three times.

*Quantitative GC Analysis*: The sampling bag was shaken up before using micro‐syringe to sample 250 µL gas (*V*_sample_) for ten times. The R22/R134a concentrations (*C*_ppm_) were calculated using Equations (1) and (2) (*AV* refers to average peak area), respectively. The relative peak area records, calculations, and summary were listed in Table S4—S6 (Supporting Information).

Equation 1 for R22:

(1) $$\;C_{\mathrm{ppm}}=R_{R22}\;\frac{\left(1.81\times 10^{-7}AV+4.08\times 10^{-6}\right)\frac{v_{\mathrm{He}}t_{\mathrm{He}}}{V_{\mathrm{sample}}}}{v_{\mathrm{gas}}t_{\mathrm{gas}}}\times 10^{6}$$

Equation 2 for R134a:

(2) $$\;C_{\mathrm{ppm}}=R_{R134a}\;\frac{\left(1.75\times 10^{-7}AV+6.37\times 10^{-7}\right)\frac{v_{\mathrm{He}}t_{\mathrm{He}}}{V_{\mathrm{sample}}}}{v_{gas}t_{gas}}\times 10^{6}$$

### Theoretical Calculations

*Adsorption Site Simulations*: First principle density functional theory (DFT) calculations were performed with the Forcite and Dmol^3^ modules of Materials Studio.^[^ ^25^ ^]^ The primitive cell of DUT‐67^[^ ^17^ ^]^ was used to reduce the size of the system for calculations. After transformed to *P*1 space group, hydrogen atoms were added to the Zr cluster for charge balance and the coordinates of all Zr atoms were fixed. Then this initial structure was first optimized using Forcite module with universal force field (UFF). The convergence tolerances of energy, force and displacement were 2.0 × 10^−5^ kcal mol^−1^, 1 × 10^−3^ kcal mol^−1^ Å^−1^, and 1 × 10^−5^ Å, respectively. Then Dmol^3^ module was used for DFT calculations. The Perdew—Burke–Ernzerhof (PBE) functional was used under the generalized gradient approximation (GGA) functional with the double‐ξ numerical polarization (DNP) basis set.^[^ ^26^ ^]^ The tolerances of energy, gradient, and displacement convergence were 1.0 × 10^−5^ hartree, 2 × 10^−3^ hartree Å^−1^, and 5 × 10^−3^ Å, respectively. Thus optimized DUT‐67 primitive cell was obtained. R22 and R134a molecules were directly optimized with Dmol^3^ using the previously described setting.^[^ ^27^ ^]^ Then these gas molecules were put into the DUT‐67 primitive cell to find out the adsorption locations. The adsorption energies (Δ*E*) of gas molecules within DUT‐67 were calculated by Δ*E* = *E*_gas_ + *E*_MOF_ − *E*_gas‐MOF_, where *E*_gas_, *E*_MOF_, and *E*_gas‐MOF_ are the total energies of the gas molecules, the MOFs, and MOF‐gas systems at their optimized geometries, respectively.^[^ ^28^ ^]^ The results are listed in Table S8 (Supporting Information).

*Grand Canonical Monte Carlo Simulations*: The grand canonical Monte Carlo (GCMC) simulations were performed with the Sorption module of Materials Studio.^[^ ^29^ ^]^ The partial charges of R22 and R134a were calculated using the ESP method.^[^ ^27^ ^]^ The previously described Dmol^3^ optimized DUT‐67 primitive cell was used. The partial charges of DUT‐67 were calculated with the charge equilibration method (Qeq) method.^[^ ^30^ ^]^ Atoms in DUT‐67 were fixed during GCMC simulations. The density distribution of the gases on the MOFs were calculated using the fixed pressure task mode of Sorption module under 298K and 100 kPa (R22 fugacity: 98.43 kPa; R134a fugacity: 97.94 kPa). The equilibration steps, production steps and temperature were 1 × 10^6^, 1 × 10^7^, and 298 K, respectively. The parameters of Zr atom was taken from UFF while others were from DREIDING force field.^[^ ^28^, ^31^ ^]^ The van der Waals interactions with a cutoff 18.5 Å were depicted by the Lennard‐Jones potential.

## Conflict of Interest

The authors declare no conflict of interest.

## Author Contributions

All authors have given approval to the final version of the manuscript.

## Supporting information

Supporting Information

Supporting Information

## Data Availability

The data that support the findings of this study are available in the supplementary material of this article.
