# Supplementary material for: Capture Fluorocarbon and Chlorofluorocarbon from Air Using DUT‐67 for Safety and Semi‐Quantitative Analysis
Source: Adv Sci (Weinh). 2024 Jan 19;11(13):2308123. doi: 10.1002/advs.202308123 (PMC10987145; doi:10.1002/advs.202308123)
Supplement: Supplementary file 1 — Supporting Information [file ADVS-11-2308123-s001.pdf]

ADVANCED SCIENCE
Open Access

Supporting Information

for *Adv. Sci.*, DOI 10.1002/advs.202308123

Capture Fluorocarbon and Chlorofluorocarbon from Air Using DUT-67 for Safety and Semi-Quantitative Analysis

Xiao-Hong Xiong, Liang Song, Wei Wang, Hui-Ting Zheng, Liang Zhang, Liu-Li Meng, Cheng-Xia Chen, Ji-Jun Jiang, Zhang-Wen Wei* and Cheng-Yong Su**

Supporting Information

Capture Fluorocarbon and Chlorofluorocarbon from Air Using DUT-67 for Safety and Semi-Quantitative Analysis

Xiao-Hong Xiong, Liang Song, Wei Wang, Hui-Ting Zheng, Liang Zhang, Liu-Li Meng, Cheng-Xia Chen*, Ji-Jun Jiang, Zhang-Wen Wei*, and Cheng-Yong Su*

MOE Laboratory of Bioinorganic and Synthetic Chemistry, GBRCE for Functional Molecular Engineering, LIFM, IGCME, School of Chemistry, Sun Yat-Sen University, Guangzhou 510006, China

Table of Contents

S1. Materials and Instrumentation 2
S2. MOF Synthesis 3
S3. Single-Crystal X-Ray Crystallography 3
S4. Gas Adsorption 8
S5. Adsorption Kinetics 11
S6. Calculations of Adsorption Isosteric Heats 12
S7. Breakthrough Experiments 14
S8. Semi-Quantitative Analysis of Low Concentration R22/R134a 15
S9. *In-Situ* SCXRD Analysis 18
S10. Theoretical Calculations 18
S11. Infrared Spectroscopy 21
S12. References 21

S1. Materials and Instrumentation

N,N'-Dimethylformamide (DMF), *N,N'*-Dimethylacetamide (DMAC), anhydrous formic acid (FC) , anhydrous methanol and anhydrous acetone were obtained from Aladdin; zirconium oxychloride octahydrate ($ZrOCl_2 \cdot 8H_2O$) and *2,5*-thiophenedicarboxylic acid were obtained from Sigma-Aldrich Co. All starting materials and solvents, unless otherwise specified, were used without further purification.

Solid-state IR spectra were recorded using Nicolet/Nexus-670 FT-IR spectrometer in the region of 4000-400 cm^{-1} using KBr pellets. Single crystal X-ray diffraction data were collected on an Agilent Technologies SuperNova X-RAY diffractometer system equipped with a Cu sealed tube (λ = 1.54178) at 50 kV and 0.80 mA. Powder X-ray diffraction (PXRD) was carried out with a RigakuSmartLab diffractometer (Bragg-Brentano geometry, Cu Kα1 radiation, λ = 1.54056 Å). Variable-temperature-dependent powder X-Ray diffraction date were collected on a RigakuSmartLab diffractometer (Bragg-Brentano geometry, Cu Kα1 radiation, λ = 1.54056 Å) under air atmosphere. *In-situ* diffuse reflectance infrared Fourier transform (DRIFT) spectra were analyzed on Thermo Fisher Nicolet IS20 spectrometer equipped with a high-sensitive Harric detector as well as *in-situ* diffuse reflectance cell (Harrick), the MCT detector was cooled down by liquid N_2 (77 K). Then the gas cell was cooled to desired reaction temperature for collecting background spectra under N_2 environment. collecting sample spectra, background spectra were subtracted, which were collected on 32 co-added scans with 4 cm^{-1} resolution. Thermogravimetric analyses (TGAs) were performed on a NETZSCH TG209 system in nitrogen and under 1 atm of pressure at a heating rate of 10 °C min^{-1}. Gas adsorption isotherms for pressures in the range of 0-1.0 bar were obtained by a volumetric method using a Quantachrome autosorb-iQ2-MP gas adsorption analyzer. Adsorption kinetic isotherms were obtained by the Vacuum Vapor/Gas Sorption Analyzer (BSD-VVS). High pressure gas adsorption isotherms for pressures in the range of 0-4.5 bar for 273K, 0-6.3 bar for 298K were obtained by BELSORP-HP high pressure gas adsorption instrument. All gas adsorption measurements were performed using ultra-high purity N_2, O_2, CO_2, R22, and R134a

gases. Breakthrough experiments were collected by two different instruments, BSD-MAB (Multi-component Adsorption Breakthrough Curve Analyzer) with mass spectrometer as detector, and a self-built instrument with gas chromatography (FL-9790 plus) as detector.

S2. MOF Synthesis

DUT-67 was synthesized with a modified literature procedure.[1]

The modulator has been changed from acetic acid to formic acid (FA) and the metal-ligand ratio has been optimized. $ZrOCl_2 \cdot 8H_2O$ (100 mg, 0.372 mmol), *2,5*-thiophenedicarboxylic acid (50 mg, 0.348 mmol), formic acid (5 mL) and DMAC (8 mL) were added into a screw-capped glass jar. The mixture was sonicated for 10 minutes then heated in a 120 ºC oven for 72 h. Cube-shaped colorless crystals appear on the wall of the glass jar. After cooling in air to room temperature, the resulting crystals were filtered and repeatedly washed with DMF.

S3. Single-Crystal X-Ray Crystallography

The single-crystal of DUT-67-R22 or DUT-67-R134a was picked and coated in para tone oil, attached to a glass silk which was inserted in a stainless-steel stick, then transferred to the Agilent Gemini S Ultra CCD Diffractometer with the Enhance X-ray Source of Cu radiation (λ = 1.54178 Å) using the ω-ϕ scan technique. All of the structures were solved by direct methods and refined by full-matrix least squares against F^2 using the SHELXL programs.[2] Hydrogen atoms were placed in geometrically calculated positions and included in the refinement process using riding model with isotropic thermal parameters: Uiso(H) = 1.2 Ueq(-CH). All the electrons of disordered solvent molecules which cannot be determined, are removed by SQUEEZE routine of PLATON program.[3] Crystal data and refinement parameters are listed in Table S7.

Note for DUT-67-R22 Refinement

The R22 molecules are disordered in pores. Thus, the occupancy of F1, F2, Cl1 and H1 were refined as 33.33%, F3 and F4 were refined as 8.33% and 4.17%, Cl2 and H5 were refined as 25% and 4.17%. C4 and C5 were refined as 33.33% and 4.17%. while the occupancy of other set of atoms was 100%. AFIX and DFIX were used to restrain the atoms. ISOR, DELU and SIMU were used to restrain the ADP refinement. The 97 restraints caused 149 refine parameters. The large pores contain highly disordered gas molecules which cannot be determined. SQUEEZE treatment was applied and the squeezed void volume is 29964 Å^3, equivalent to 51.7% of the unit cell. The R1 value is 0.113 without SQUEEZE treatment and 0.0732 with SQUEEZE treatment.

Note for DUT-67-R134a Refinement

The R134a molecules are disordered in pores. Thus, the occupancy of F1, F2, F3, F4, C4, C5, H5A and H5B were refined as 16.67%. while the occupancy of other set of atoms was 100%. AFIX, DFIX and SADI were used to restrain the atoms. ISOR, DELU and SIMU were used to restrain the ADP refinement. The 118 restraints caused 140 refine parameters. The large pores contain highly disordered gas molecules which cannot be determined. SQUEEZE treatment was applied and the squeezed void volume is 31360 Å^3, equivalent to 53.5% of the unit cell. The R1 value is 0.1087 without SQUEEZE treatment and 0.0665 with SQUEEZE treatment.

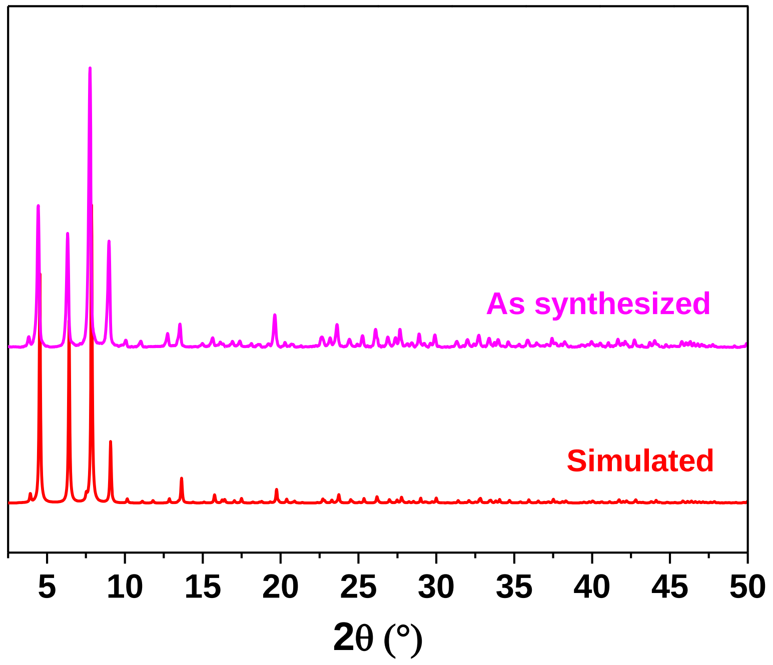

Figure S1. The PXRD patterns of DUT-67.

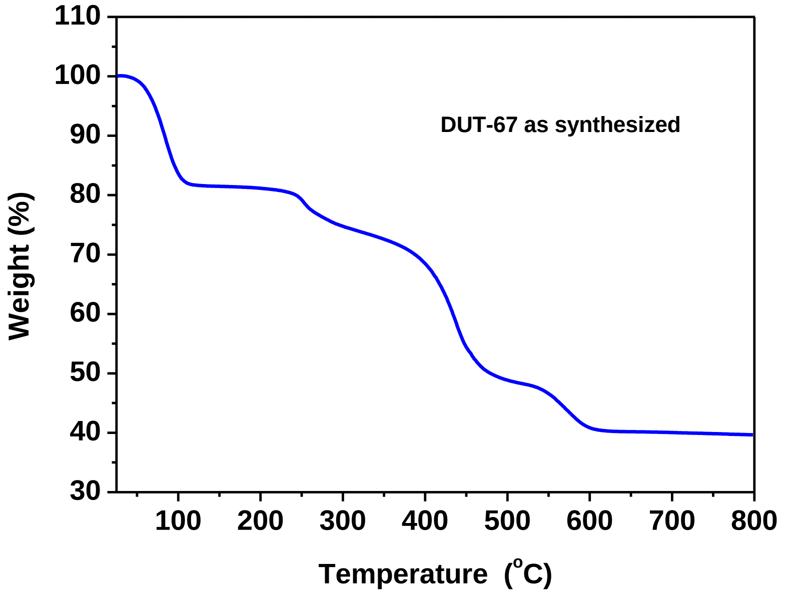

Figure S2. Thermogravimetric analyses (TGAs) of the fresh DUT-67 in nitrogen and under 1 bar of pressure at a heating rate of 10 °C min^{-1}.

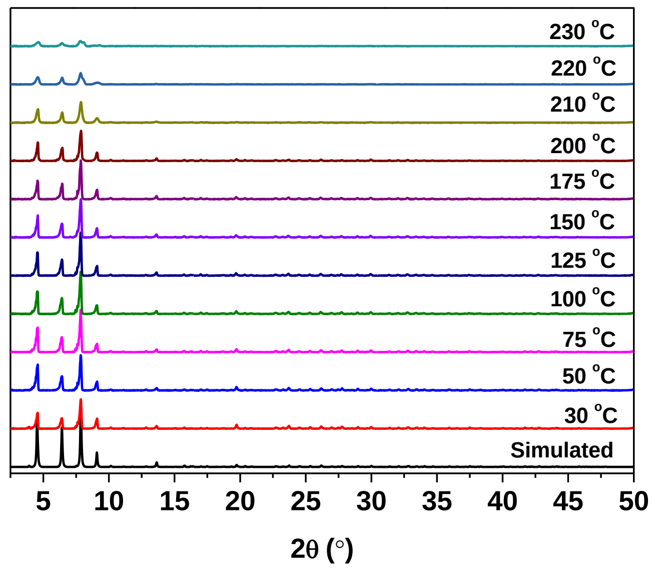

Figure S3. The variable-temperature PXRD patterns of DUT-67 in air and under 1 bar of pressure.

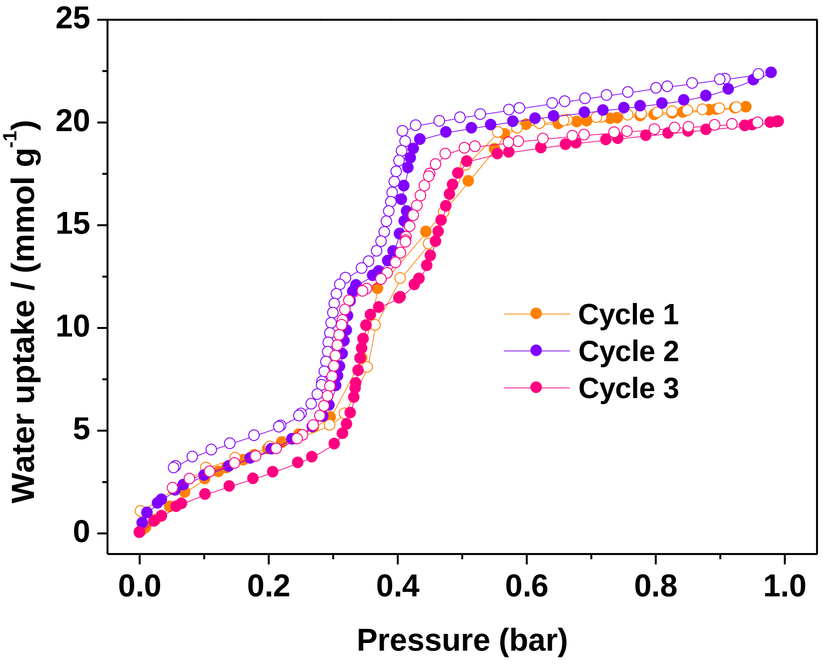

Figure S4. Three cycles of water adsorption isotherms of DUT-67 at 298 K (After each test finished, the sample was activated at 100 °C for 4 hours).

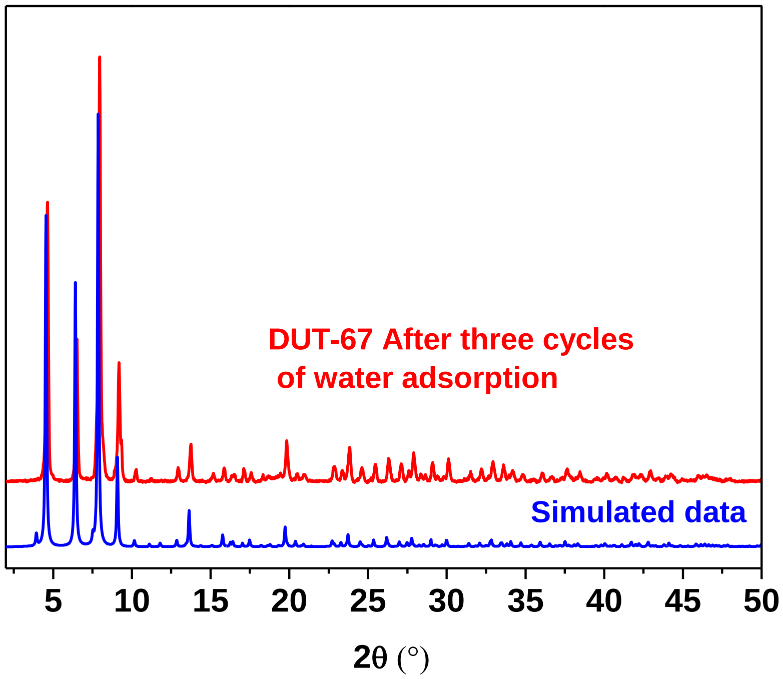

Figure S5. The PXRD patterns of DUT-67 after three water adsorption isotherms.

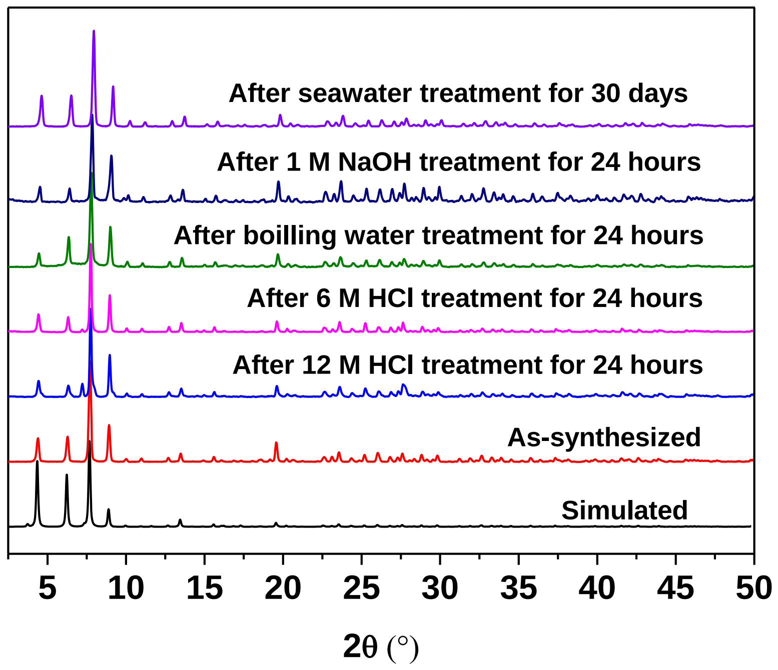

Figure S6. The PXRD patterns of DUT-67 after soaking in various aqueous solutions for different times.

S4. Gas Adsorption

N_2 Sorption Isotherm at 77 K: Before gas sorption experiments, as-synthesized DUT-67 was washed with 3×10 mL DMF and immersed in DMF for 2 days. During this period, the DMF was replaced three times per day. Then the DMF-exchanged sample was filtrated off and immersed in anhydrous acetone for 3 days, during which the solvent was decanted and freshly replenished 3 times every day. The solvent-exchanged samples were activated under vacuum at 60 °C for 4 h and 120 °C for 12 h. Gas sorption measurements were then conducted using a Quantachrome autosorb-iQ2-MP gas adsorption analyzer. The BET calculation fittings and pore size distributions are plotted in Figure S7 and Figure S8, respectively.

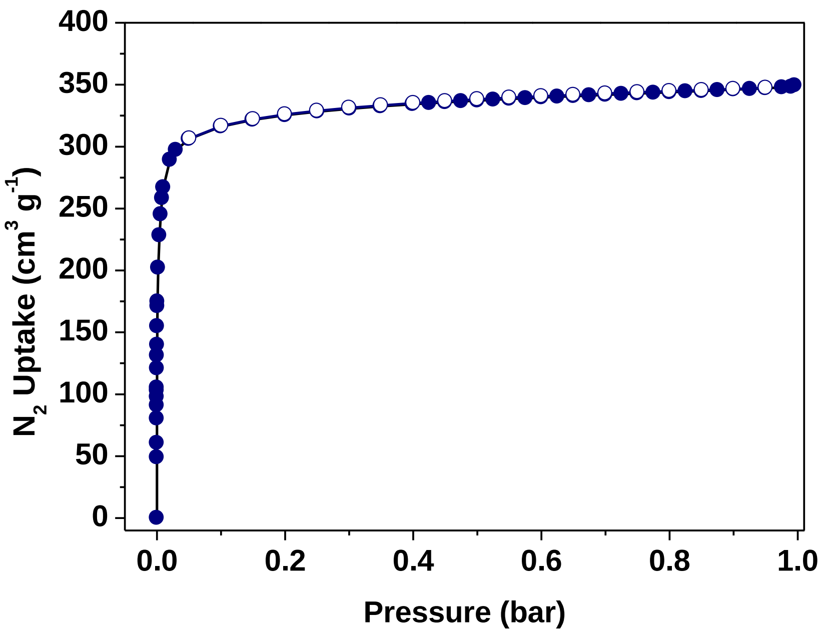

Figure S7. N_2 adsorption isotherm of DUT-67 at 77 K.

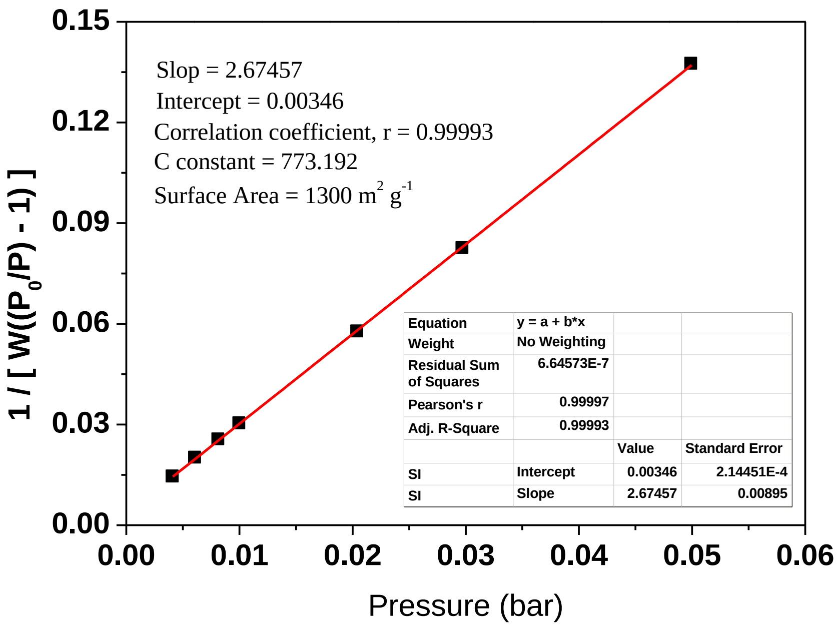

Figure S8. Plot of the linear region on the N_2 isotherm of DUT-67 for the BET equation.

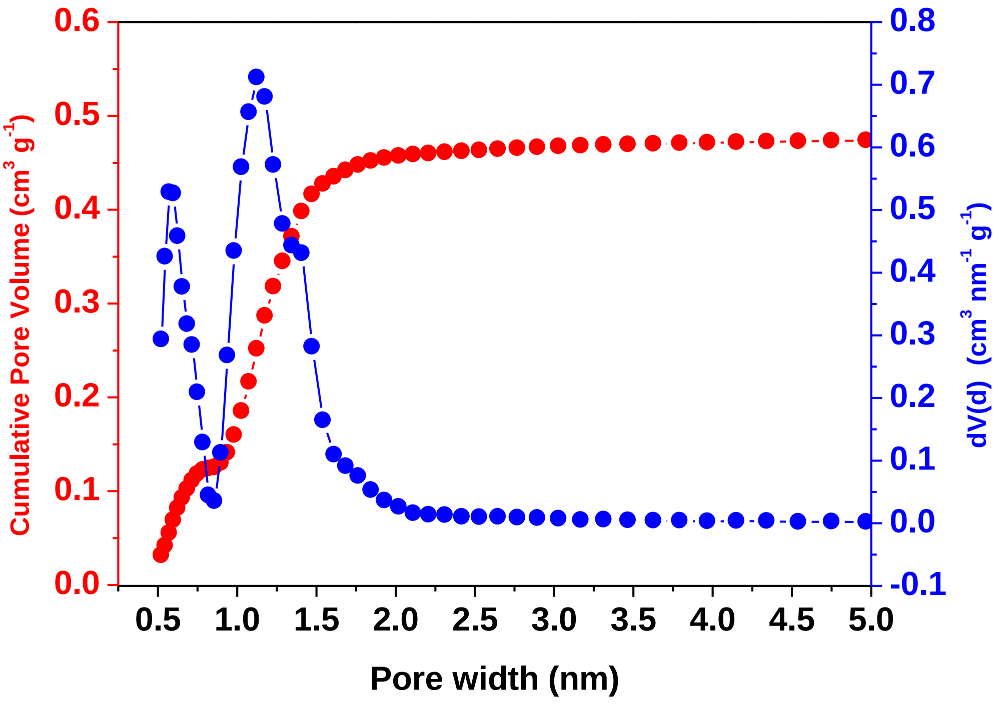

Figure S9. Pore size distribution of DUT-67 calculated from DFT analysis. Calculation model: N_2 at 77 K on carbon (slit/cylindr./sphere pores, NLDFT adsorption branch). Fitting error: 0.377 %.

Table S1 Summary of reported MOFs and molecule sieves with BET surface areas, R22/R134a gas uptake capacities, and adsorption enthalpy of R22 and R134a.

Adsorbent	BET surface area (m^2 g^{-1})	R22 uptake (cm^3 g^{-1})	R134a uptake (cm^3 g^{-1})	R22 Q_{st} (kJ mol^{-1})[a]	R134a Q_{st} (kJ mol^{-1})[b]	Ref.
DUT-67	1300	124	116	42.3	49.3	This work
UiO-66(Zr)	1383	112	-	--	31	4
MAF-X10	2032	70.9	-	32.9	-	5
MAF-X12	1787	63.1	-	31.8	-	
MAF-X13	2742	85.51	-	31.4	-	
LIFM-28np	940	69 [c]	-	26	-	6
LIFM-29	1482	136.1 [c]	-	27	-	
LIFM-30	1306	139.2 [c]	-	36.5	-	
LIFM-31	1307	158.6 [c]	-	26.7	-	
LIFM-32	1472	110.5 [c]	-	33.6	-	
LIFM-33	1588	82.7 [c]	-	33.9	-	

LIFM-82	1624	196.4 c	-	31.3	-	7
LIFM-86	1269	139.0c	--	30.4	-	
LIFM-26	1513	145c	-	25	-	8
Ni-MOF-74	1146	-	127	-	-	9
Ni-BPP	2039	-	162	-	-	
Ni-TPP	1975	-	166	-	-	
Mesh 4A	626	35.8d	-	-	-	10
Mesh 5A	484	62.72d	-	-	-	
LIFM-66	916	220	239	27.9	35.6	11
LIFM-66/67-mix	836	215	241	36.1	37.8	
LIFM-67	819	249.8	249.5	36.2	38.9	
MCF-61	2096	-	188.9	-	30	12
MCF-62	2630	-	169	-	29.3	
MCF-63	2749	-	166.8	-	28.8	
MOFF-5	2445	-	120.7	-	-	13
PCN-222	169	-	149.3	-	-	14
NU-1000	2259	-	175.63	-	-	

[a] Q_{st} values at zero coverage;

[b] for 10/90 (v/v) $R22/CO_2$ or $R134a/CO_2$ mixture at 298 K and zero coverage;

[c] uptakes under 273 K;

[d] uptakes under 293 K;

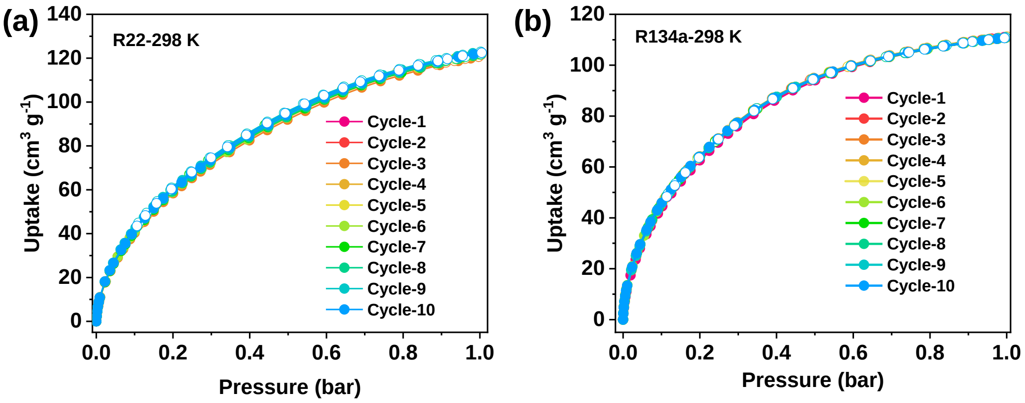

Figure S10. (a) R22 and (b) R134a cycling adsorption tests of DUT-67 under 298 K.

S5. Adsorption Kinetics

The adsorption kinetics was analyzed by simplified kinetic models such as the pseudo first order and pseudo second order, through the following two equations. The calculation and fitting results show that the linear correlation coefficient (R^2) of the

pseudo first order is very low, so we choose the pseudo-second-order mode to calculate the sorption rate constant of R22 and R134a.

$$\frac{t}{q_t} = \frac{1}{k_2 q_e} + \frac{t}{q_e} \qquad \text{(Pseudo second order mode)}$$

Where q_e (mg g^{-1}) and q_t (mg g^{-1}) are the quantity of the R22 and R134a adsorbed at equilibrium and at t time, respectively, and the k_2[g (mg min)$^{-1}$] is the pseudo second order sorption rate constant that is deduced from the slope of the plot of t/q_t versus t.

Table S2. Kinetic parameters of quasi-second order dynamics of R22 and R134a adsorption by DUT-67.

	R134a				R22			
Pressure	q_e, exp, (cm^3 g^{-1})	q_e, cal (cm^3 g^{-1})	k_2 (g·mg^{-1} min^{-1})	R^2	q_e, exp (cm^3 g^{-1})	q_e, cal (cm^3 g^{-1}g^{-1})	k_2 (g·mg^{-1} min^{-1})	R^2
0.05	96.1797	96.7117	0.08742	0.9999	65.8359	66.5375	0.06968	0.9998
0.1	148.2479	148.3671	0.03452	0.9999	104.4599	108.4598	0.03058	0.9997
0.2	224.4571	230.4142	0.02125	0.9999	172.117	164.9023	0.01998	0.9992
0.4	309.709	316.4506	0.01639	0.9998	236.9013	247.524	0.01509	0.9988
0.6	348.8602	357.1244	0.01555	0.9998	284.1435	290.6977	0.01675	0.9996
0.8	371.208	383.1423	0.01133	0.9997	312.1127	319.4881	0.01486	0.9996
1	383.6691	392.1568	0.01214	0.9993	328.3809	336.7321	0.01292	0.9995

S6. Calculations of Adsorption Isosteric Heats

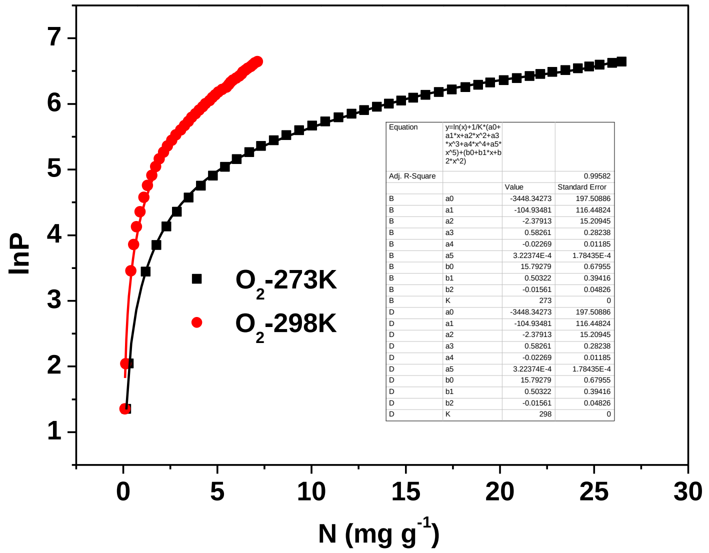

Figure S11. O_2 virial fitting (lines) of the adsorption isotherms (points) of DUT-67 measured at 273 and 298 K.

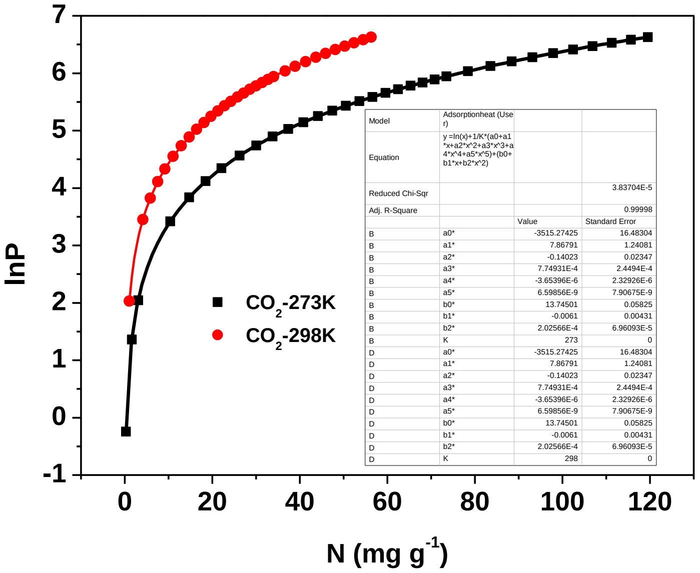

Figure S12. CO_2 virial fitting (lines) of the adsorption isotherms (points) of DUT-67 measured at 273 and 298 K.

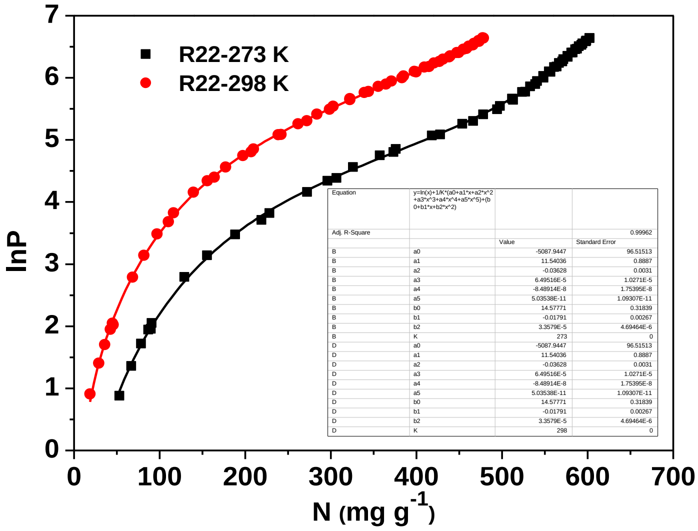

Figure S13. R22 virial fitting (lines) of the adsorption isotherms (points) of DUT-67 measured at 273 and 298 K.

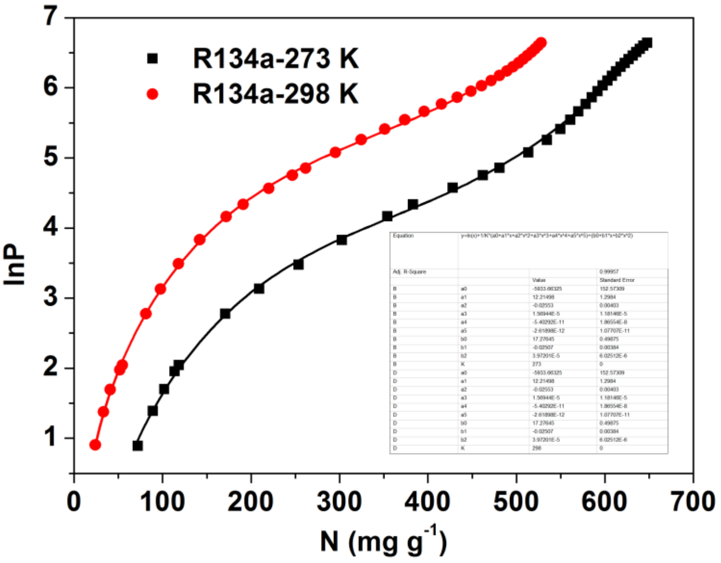

Figure S14. R134a virial fitting (lines) of the adsorption isotherms (points) of DUT-67 measured at 273 and 298 K.

S7. Breakthrough Experiments

Transient breakthrough experiments for the separation of R22/R134a/air and R22/R134a/air (1:1:98, v/v/v), R22/R134a/air (10:10:80, v/v/v) were carried out in a fixed bed. The flow rates of gases were regulated by mass flow controllers. The column (6 mm inner diameter × 150 mm) contained 1.0 g of pre-activated sample for the experiment using a binary /ternary component. Before filled in the column, the samples were activated at 333 K for 12 hours under vacuum conditions. After filling the column, the column was purged with a He flow (30 mL min^{-1} at 298 K and 1 bar) for 2 h. Then the gas mixture was introduced to the column. The outlet composition was continuously monitored by a mass spectrometry until a complete breakthrough was achieved. The sample was regenerated with a He flow (30 mL min^{-1}) at 373 K until all gases signal disappeared before each cyclic experiment. For the separation of R22/R134a/air (0.1:0.1:99.8, v/v/v) and (0.002:0.002:99.996, v/v/v) the outlet composition was continuously monitored by a gas chromatograph (FULI GC9790 Plus) until a complete breakthrough was achieved.

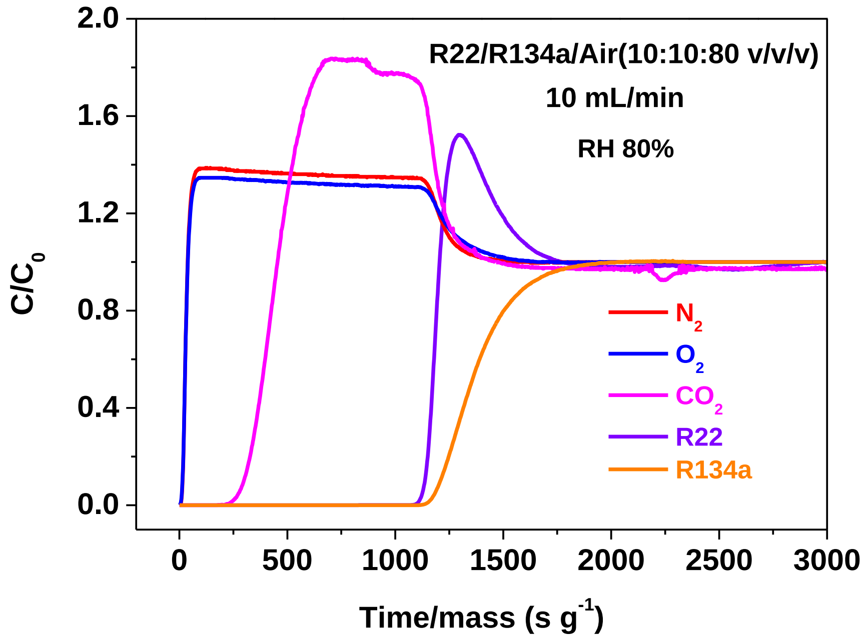

Figure S15. The column breakthrough dynamic separations curve of DUT-67 at 298 K under 80% humid condition.

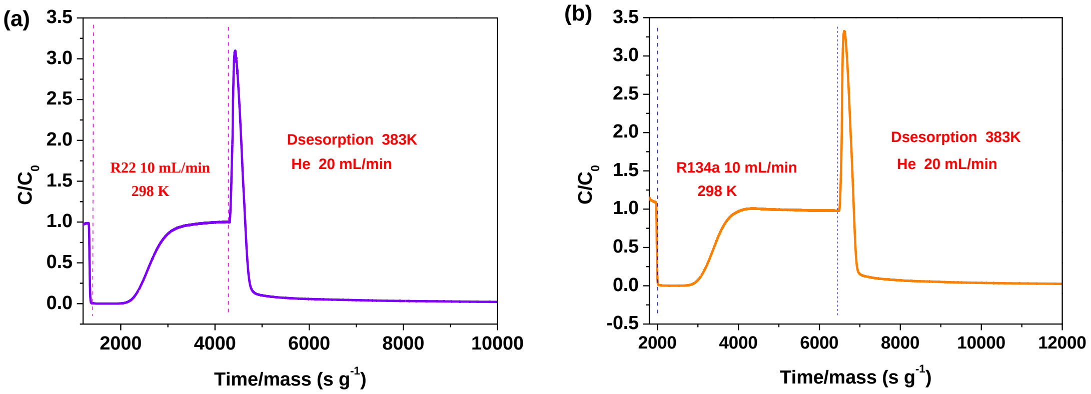

Figure S16. The capture-desorption curves of pure (a) R22 and (b) R134a.

S8. Semi-Quantitative Analysis of Low Concentration R22/R134a

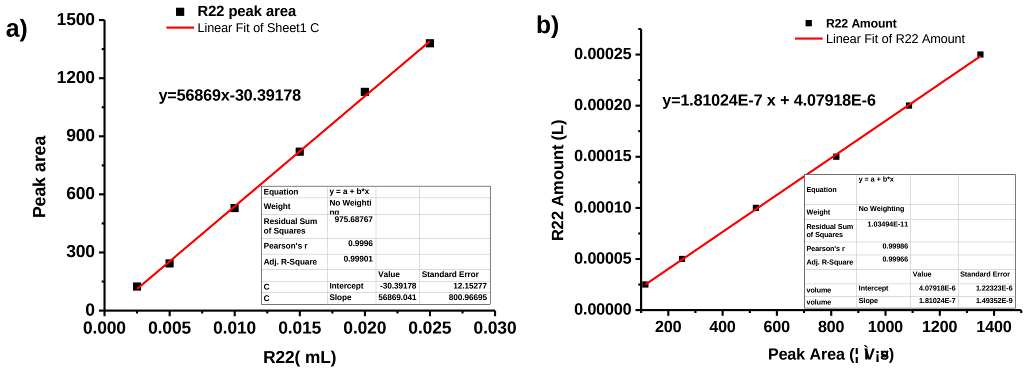

Figure S17. The working curve of R22. a) R22 injection volume vs corresponding peak area in GC, b) R22 peak area in GC vs R22 injection volume (in order to directly calculate the volume of R134a in an unknown sample).

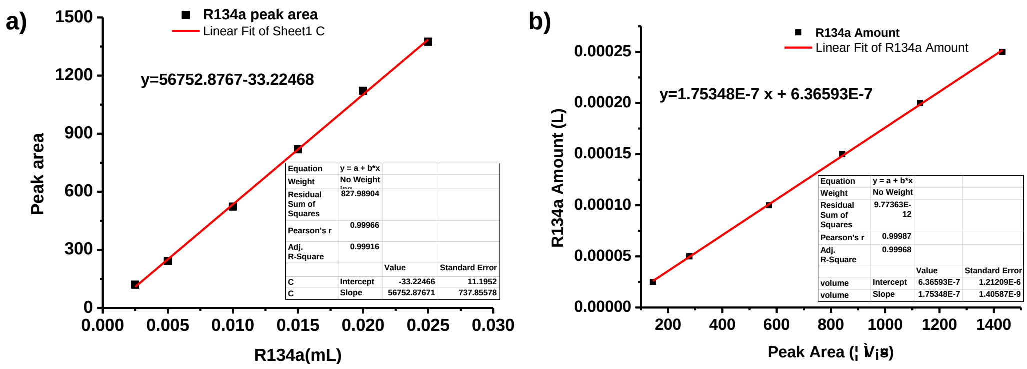

Figure S18. The working curve of R134a. a) R134 injection volume vs corresponding peak area in GC, b) R134 peak area in GC vs R134 injection volume (in order to directly calculate the volume of R134a in an unknown sample).

Limit of detection calculation (LOD): The LOD was inferred by eq 1. the LOD is defined as 3 times the standard deviation (3σ) of the zero determinations, in which the values of the sensitivity are 56869 and 56752 for R22 and R134a (see Figure S17 a and Figure S17a) [15].

$$LOD = \frac{3\theta}{Sensitivity} \quad \text{(eq 1)}$$

The LOD of this method for R22 and R134a were estimated to be 1.533 × 10^{-5} mL and 4.772 × 10^{-5} mL according to Figure S16 a and Figure S17a.

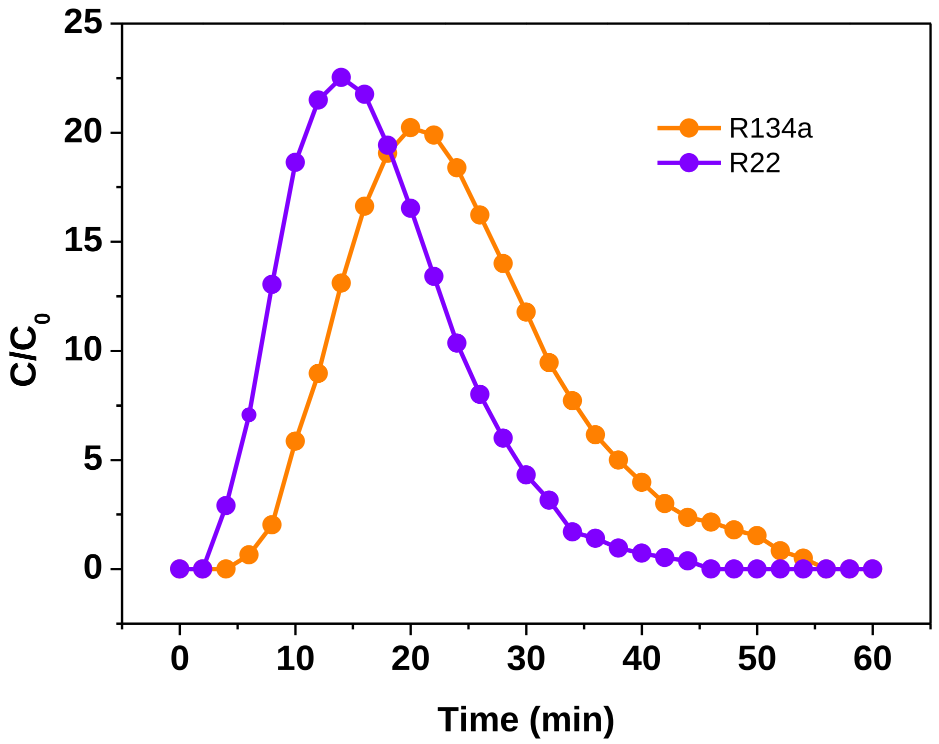

Figure S19. The desorption curve of DUT-67 samples after capturing low concentration R22/R134a.

Table S3. Calibration factors from the calculated concentrations of R22/R134a in R22/R134a/air (0.002:0.002:99.996, v/v/v) mixture after blank breakthrough process.

Gas	Peak area (48 tests)								AV	Calculated concentration (ppm)	Calibration factor (*R*)
R22	162	161	160	154	156	151	153	153	157	25.99	0.77
	167	151	151	153	168	161	166	151			
	157	146	168	149	154	148	146	157			
	151	161	159	154	160	153	169	167			
	168	151	150	165	150	165	139	160			
	156	161	156	161	163	161	156	166			
R134a	162	147	156	163	149	160	152	151	150	21.65	0.92
	158	145	140	155	154	149	160	163			
	142	160	141	152	141	156	150	155			
	140	152	146	157	139	152	153	143			
	155	144	152	142	142	145	140	155			
	148	151	152	152	146	153	152	140			

Table S4. Relative peak area records and calculations of R22 concentrations in the enriched R22/R134a/air (0.002:0.002:99.996, v/v/v) samples.

1	2	3	4	5	6	7	8	9	10	Averaged AV	C_{ppm}	Deviation
155	158	157	153	157	151	168	175	169	165	161	20.91	0.91
182	151	167	171	164	160	156	172	187	163	167	20.69	0.69

168	189	163	159	167	163	159	156	179	166	167	21.02	1.02

Table S5. Relative peak area records and calculations of R134a concentration in the enriched R22/R134a/air (0.002:0.002:99.996, v/v/v) samples.

1	2	3	4	5	6	7	8	9	10	AV	C_{ppm}	Deviation
151	161	160	154	156	151	165	164	162	167	159	21.00	1.00
169	167	167	157	176	155	146	172	164	184	166	22.08	2.08
167	171	158	187	171	164	173	166	183	166	171	22.73	2.73

Table S6. Summary of the semi-quantitative analysis results for R22 and R134a.

Gas	Average C_{ppm}	Standard deviation	Relative standard deviation (%)	Absolute Error (ppm)	Relative Error (%)
R22	20.87	0.17	0.83	0.87	4.17
R134a	21.93	0.87	3.98	1.93	8.80

S9. *In-Situ* SCXRD Analysis

Table S7. Crystal data and structure refinement for DUT-67-R22 and DUT-67-134a.

**Compound**	**DUT-67-R22**	**DUT-67-R134a**
CCDC No.	2295117	2295116
Empirical formula	$C_{27}H_{11}Cl_3F_6O_{32}S_4Zr_6$	$C_{26.67}H_{10.67}F_{5.33}O_{32}S_4Zr_6$
Formula weight	1743.27	1619.91
Temperature/K	240.00(10)	240.00(10)
Crystal system	cubic	cubic
Space group	*Fm*-3*m*	*Fm*-3*m*
a/b/c (Å)	38.8577(2)	38.8516(2)
α/β/γ/°	90	90
Volume/Å^3	58672.1(9)	58644.4(11)
Z	24	24
ρ_{calc} (g/cm^3)	1.184	1.101
μ (mm^{-1})	7.181	6.403
F(000)	20112.0	18688.0
Crystal size (mm^3)	0.1 × 0.1 × 0.1	0.1 × 0.1 × 0.1
Radiation	CuKα (λ = 1.54184)	CuKα (λ = 1.54184)
2Θ range for data collection (°)	7.546 to 144.776	7.546 to 136.3
Index ranges	$-16 \le h \le 46$, $-32 \le k \le 7$, $-44 \le l \le 31$	$-22 \le h \le 38$, $-29 \le k \le 35$, $-46 \le l \le 44$
Reflections collected	10767	10377
Independent reflections	2820 [R_{int} = 0.0572, R_{sigma} = 0.0365]	2641 [R_{int} = 0.0299, R_{sigma} = 0.0264]
Data/restraints/parameters	2820/97/149	2641/118/140

Goodness-of-fit on F^2	1.064	1.118
Final R indexes [I>=2σ (I)]	R_1 = 0.0764, wR_2 = 0.2239	R_1 = 0.0665, wR_2 = 0.1934
Final R indexes [all data]	R_1 = 0.0809, wR_2 = 0.2331	R_1 = 0.0726, wR_2 = 0.2009
Largest diff. peak/hole / e $Å^{-3}$	1.74/-1.26	3.94/-1.48

S10. Theoretical Calculations

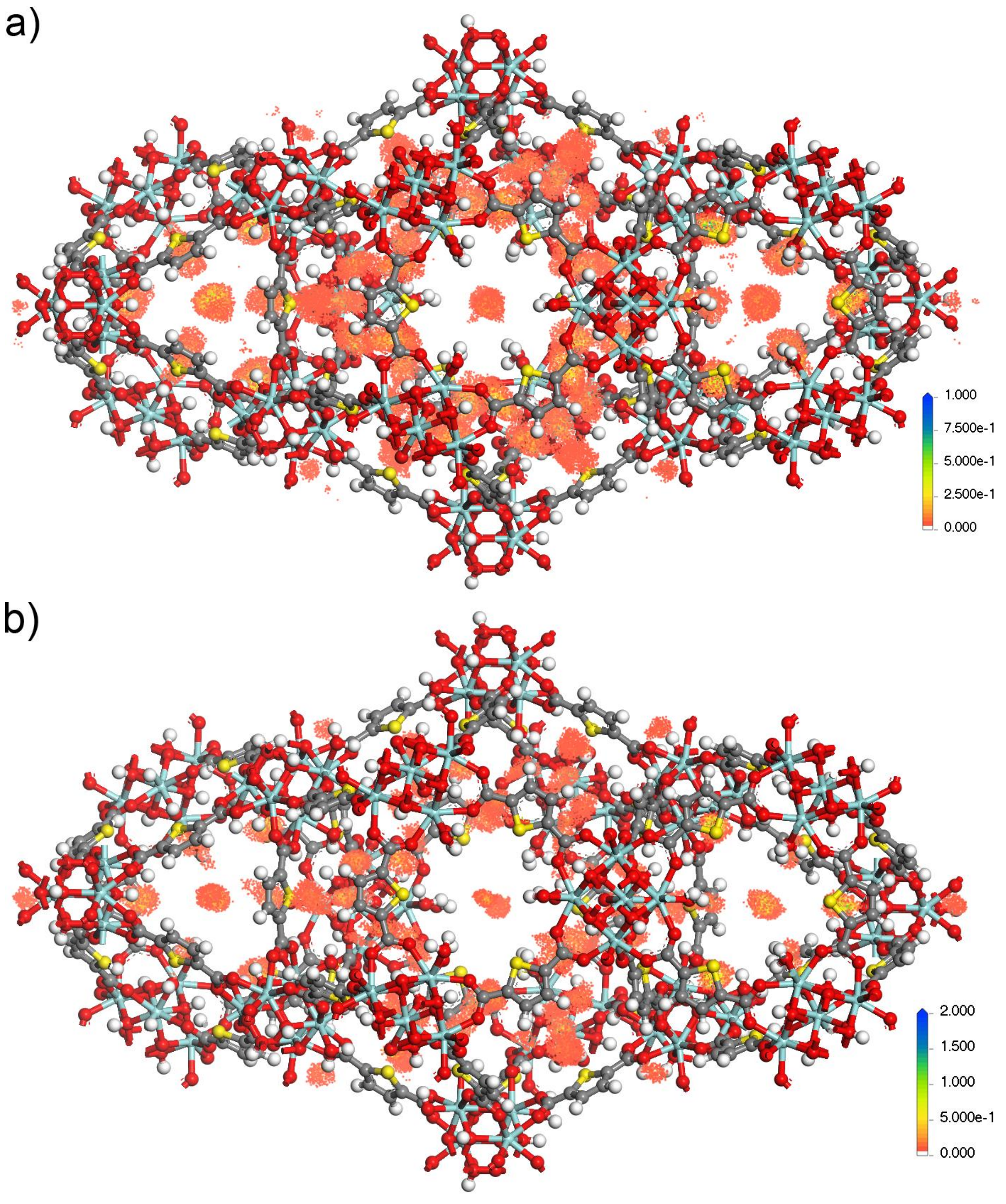

Figure S20. Density distribution of (a) R22 and (b) R134a in optimized DUT-67 at 298K and 100 kPa.

Table S8. Summary of DFT calculation results.

Systems	E (Ha)	ΔE (kJ mol^{-1})
DUT-67	-157033.9370658	N/A
R22	-698.3042115	N/A
R134a	-476.5413181	N/A
DUT-67-R22	-157732.2512968	26.31
DUT-67-R134a	-157510.4914537	34.31

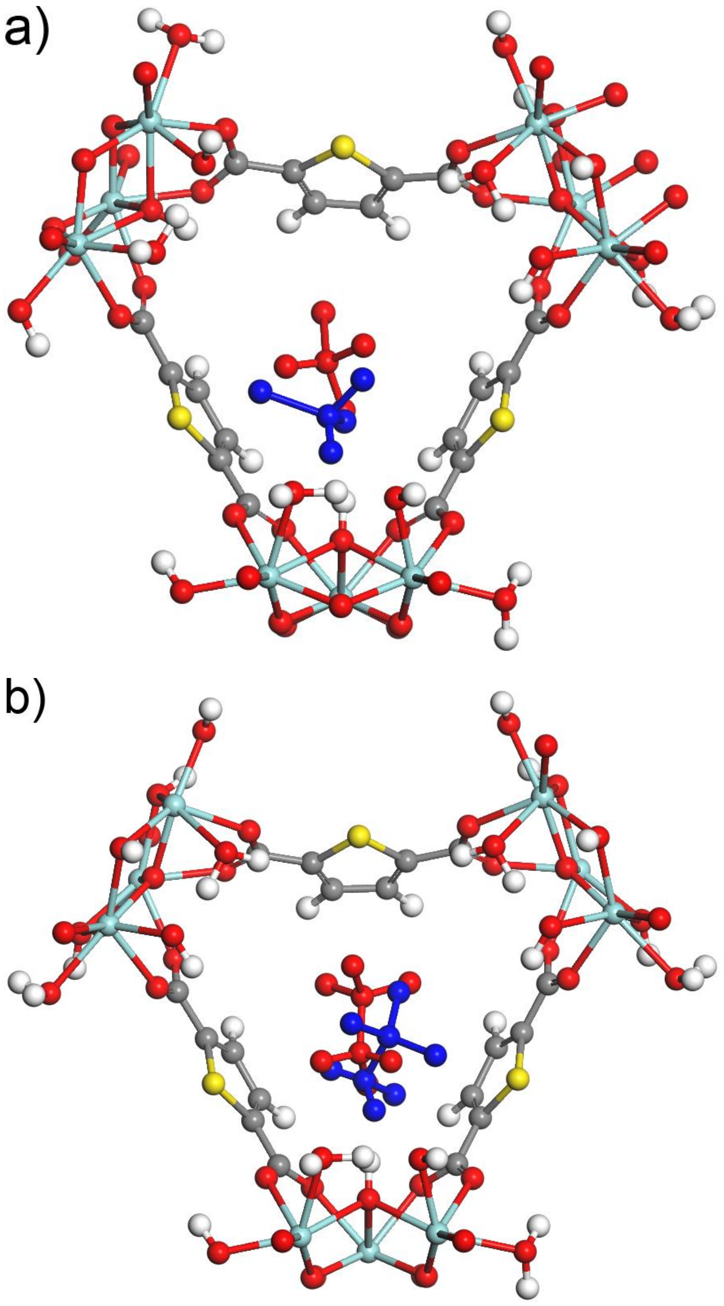

Figure S21. The single-crystal X-ray diffraction (red) and DFT calculation determined (blue) adsorption sites of (a) R22 and (b) R134a, respectively.

S11. Infrared Spectroscopy

11.1 *In-Situ* Diffuse Reflectance Infrared Fourier Transform (DRIFT) Spectra

In-situ DRIFT spectra were collected on Thermo Fisher Nicolet 6700 spectrometer with a high-sensitive MCT detector, which was cooled down by liquid N_2 (77 K). Activated DUT-67 was placed into an IR gas cell (PIKE), heated at 100 °C in a heated rate of 2 °C min^{-1} while purge with high purity N_2 gas (50 mL min^{-1}) for 40 min to remove adsorbed water on the surface of samples. Then the gas cell was cooled down to the required temperature for collecting background in high purity N_2 atmosphere. Before collecting sample spectra, background spectra were subtracted, which were collected on 32 co-added scans with 4 cm^{-1} resolution. Then pure R22 and R134a were injected into the sample cell for tests, respectively.

11.2 Infrared Spectra

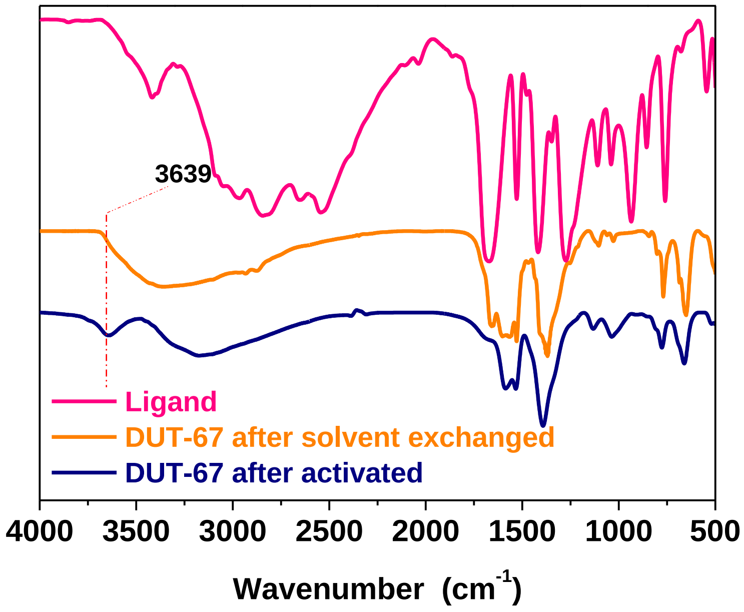

Figure S22. Infrared spectra of ligand and DUT-67 after-solvent-exchanged/activated.

S12. References

(1) Bon, V.; Senkovska, I.; Baburin, I. A.; Kaskel, S. Zr- and Hf-Based Metal–Organic Frameworks: Tracking Down the Polymorphism. *Cryst. Growth Des.* **2013**, *13*, 1231-1237.

(2) Sheldrick, G. M. A short history of SHELX. *Acta Crystallogr. A* **2008**, *64*, 112-122.

(3) Spek, A. L. PLATON SQUEEZE: a tool for the calculation of the disordered solvent contribution to the calculated structure factors. *Acta Crystallogr. C* **2015**, *71*, 9-18.

(4) Darshika, K. J. A. W.; Jiajian, G.; Bin, L., Fluorocarbon Separation in a Thermally Robust Zirconium Carboxylate Metal–Organic Framework. *Chem. Asian J.* **2018,** *13* (8), 977-981.

(5) Lin, R. B.; Li, T. Y.; Zhou, H. L.; He, C. T.; Zhang, J. P.; Chen, X. M., Tuning Fluorocarbon

Adsorption in New Isoreticular Porous Coordination Frameworks for Heat Transformation Applications. *Chem. Sci.* **2015,** *6*, 2516-2521.
(6) Chen, C. X.; Wei, Z.; Jiang, J. J.; Fan, Y. Z.; Zheng, S. P.; Cao, C. C.; Li, Y. H.; Fenske, D.; Su, C. Y., Precise Modulation of the Breathing Behavior and Pore Surface in Zr-MOFs by Reversible Post-Synthetic Variable-Spacer Installation to Fine-Tune the Expansion Magnitude and Sorption Properties. *Angew. Chem. Int. Ed.* **2016,** *55* (34), 9932-9936.
(7) Chen, C. X.; Wei, Z. W.; Jiang, J. J.; Zheng, S. P.; Wang, H. P.; Qiu, Q. F.; Cao, C. C.; Fenske, D.; Su, C. Y., Dynamic Spacer Installation for Multirole Metal-Organic Frameworks: A New Direction toward Multifunctional MOFs Achieving Ultrahigh Methane Storage Working Capacity. *J. Am. Chem. Soc.* **2017,** *139* (17), 6034-6037.
(8) Chen, C. X.; Zheng, S. P.; Wei, Z. W.; Cao, C. C.; Wang, H. P.; Wang, D.; Jiang, J. J.; Fenske, D.; Su, C. Y., A Robust Metal-Organic Framework Combining Open Metal Sites and Polar Groups for Methane Purification and CO_2/Fluorocarbon Capture. *Chem. Eur. J.* **2017,** *23* (17), 4060-4064.
(9) Jian, Z.; Rama, S. V.; Luis, E.; Phillip, K. K.; Tamas, V.; Donald, M. C.; Thomas, A. B.; McGrail, B. P.; Radha Kishan, M., Pore-Engineered Metal–Organic Frameworks with Excellent Adsorption of Water and Fluorocarbon Refrigerant for Cooling Applications. *J. Am. Chem. Soc.* **2017,** *139* (31), 10601–10604.
(10) Darshika, K. J. A. W.; Jiajian, G.; Tetsuo, T.; Qichun, Z.; Bin, L., Adsorption Separation of R-22, R-32 and R-125 Fluorocarbons Using 4A Molecular Sieve Zeolite. *ChemistrySelect* **2016,** *1* (13), 3718-3722.
(11) Xiong, Y.-Y.; Krishna, R.; Pham, T.; Forrest, K. A.; Chen, C.-X.; Wei, Z.-W.; Jiang, J.-J.; Wang, H.-P.; Fan, Y.; Pan, M.; Su, C.-Y., Pore-Nanospace Engineering of Mixed-Ligand Metal–Organic Frameworks for High Adsorption of Hydrofluorocarbons and Hydrochlorofluorocarbons. *Chem. Mater.* **2022,** *34* (11), 5116-5124.
(12) Mo, Z. W.; Zhou, H. L.; Zhou, D. D.; Lin, R. B.; Liao, P. Q.; He, C. T.; Zhang, W. X.; Chen, X. M.; Zhang, J. P., Mesoporous Metal-Organic Frameworks with Exceptionally High Working Capacities for Adsorption Heat Transformation. *Adv. Mater.* **2018,** *30* (4), 1704350.
(13)Chen, T. H.; Popov, I.; Kaveevivitchai, W.; Chuang, Y. C.; Chen, Y. S.; Jacobson, A. J.; Miljanic, O. S., Mesoporous Fluorinated Metal-Organic Frameworks with Exceptional Adsorption of Fluorocarbons and CFCs. *Angew. Chem Int. Ed.* **2015,** *54* (47), 13902-13906.
(14) Zheng, J.; Barpaga, D.; Gutiérrez, O. Y.; Browning, N. D.; Mehdi, B. L.; Farha, O. K.; Lercher, J. A.; McGrail, B. P.; Motkuri, R. K., Exceptional Fluorocarbon Uptake with Mesoporous Metal–Organic Frameworks for Adsorption-Based Cooling Systems. *ACS Appl. Energy Mater.* **2018,** *1*(11), 5853-5858.
(15) Lu, X. W.; Jiang, L. X.; Liu, J.; Yang, Y.; Liu, Q. Y.; Ren, Y.; Li, X.; He, S. G., Sensitive Detection of Gas-Phase Glyoxal by Electron Attachment Reaction Ionization Mass Spectrometry. *Anal. Chem*. **2019**, *91*, 12688-12695.
